# Detection of substance use in clinical forensic cases: urine analysis of victims and perpetrators

**DOI:** 10.1007/s12024-024-00873-w

**Published:** 2024-09-05

**Authors:** Pia Johansson Heinsvig, Katinka Rønnow Holler, Christian Lindholst, Trine Skov Nielsen

**Affiliations:** Department of Forensic Medicine, Section for Forensic Pathology, Aarhus University, Palle Juul-Jensens Boulevard 99, Aarhus N, 8200 Denmark

**Keywords:** Clinical forensic medicine, Toxicology, Urine, Liquid chromatography-mass spectrometry, Victim, Perpetrator

## Abstract

This study investigates the prevalence of substance use among victims and perpetrators involved in clinical forensic cases. Urine samples from 455 individuals aged 18 and above, collected in 2019, were analyzed using two LC-MS-based analytical methods and an HS-GC-FID method for the most frequently reported substances of abuse and medication. Data from case documents, encompassing gender, age, and the individual’s role, were recorded in a database. Both the urine samples and the information from case documents were fully anonymized. The most frequently detected substance was alcohol (37% of all cases), followed by cannabis (22% of all cases) and central nervous system stimulants (24% of all cases). Other classes of substances detected included benzodiazepines, anabolic steroids, antipsychotic agents, and antidepressants. No drugs or alcohol were detected in 32% of the victims and 19% of the perpetrators. The study also examines the interrelationship of drug patterns between victims and perpetrators, and results show that both parties were influenced by substances at the time of the incident. Furthermore, there was a significant difference in the use of substances between perpetrators of blunt and sharp force cases and perpetrators in cases of sexual assault. Timely sample collection and a structured toxicological analysis of both victims and perpetrators in the same case are vital in clinical forensic cases to enhance comprehension of the connection between criminal activities and substance use. This understanding enables the development of prevention strategies at an informed level.

**Supplementary Information:**

The online version contains supplementary material available at 10.1007/s12024-024-00873-w.

## Introduction

Excessive alcohol and drug use remains a pressing public health issue with far-reaching consequences for society. These include criminal activity, higher medical expenses, and negative social impact on individuals, families and communities [1–3]. Understanding the specific types of drugs being used and the magnitude of their effects allows for targeted interventions and policies aimed at mitigating the harms associated with substance abuse.

Recent research has increasingly linked drugs and alcohol abuse with violent behavior [4–6]. Studies indicate that those who engage in criminal activities report higher levels of drug and alcohol use compared to non-criminals. Additionally, individuals who use alcohol and illegal drugs are more likely to engage in criminal behavior than non-users [6, 7]. Substances such as central nervous system (CNS) stimulants, cannabis, anabolic steroids, and opioids have been linked to violent behavior [5, 7, 8]. Cocaine, for instance has been associated with both perpetrators and victims in blunt force and sharp force cases [9, 10]. Methamphetamine use has been correlated with violent behavior in blunt force cases, though no clear correlation has been established between drug ude and the type of criminal activities [11, 12].

The majority of published studies examining biological samples from victims in clinical forensic cases focus on two types of cases: sexual assault and homicide/suicide. ElSohly et al. conducted a study of 1,179 alleged victims of sexual assault in 1999 and found that alcohol had the highest prevalence, detected in 41% of cases. Alcohol, in addition to its direct harm to the user, has the highest negative impact on individuals other than the user [13–15]. Other studies also found alcohol to be the most commonly involved substance in sexual assault cases for both victims and perpetrators [13, 14]. Substances detected in ElSohly et al.‘s study included cannabinoids, cocaine, benzodiazepines, amphetamines, and GHB. More than one substance was detected in 21% of the cases, although 39% of the samples did not include alcohol or drugs [16]. Additionally, it is well-documented that perpetrators of sexual assault tend to target individuals under the influence of alcohol or other drugs, regardless of their own substance use [14, 17, 18].

Drake et al. found that half of both perpetrators and victims of homicide had psychoactive substances in their blood, with alcohol being the most common substance detected [19]. This finding was supported by Hedlund et al., who examined victims and perpetrators of homicide in Sweden between 2007 and 2009, finding a positive toxicology in 57% of victims and 62.5% of perpetrators, with alcohol being the most frequent substance, followed by benzodiazepines [20]. Another study examined the use of anabolic steroids, the most frequently reported performance and image-enhancing drug (PIED), among perpetrators in various clinical forensic cases and driving under the influence (DUI) cases from 1999 to 2009, showing that 30% of steroid users had a higher prevalence of drug abuse compared to non-steroid users [4].

Studies on substance abuse in other types of criminal cases, such as blunt force trauma, sharp force trauma, and shootings, are sparse. Understanding the relationship between these different types of criminal acts may provide insights for more effective prevention strategies. In the present study, 455 urine samples collected from both victims and perpetrators in 2019 were analyzed for the presence of drugs using an earlier-developed UPLC-HR-qTOF-MS method [21]. The findings from the UPLC-HR-qTOF-MS method were verified by an LC-MS/MS method. Additionally, samples were analyzed using an HS-GC-FID method to detect alcohol. This study aims to provide insight into substance use among victims and perpetrators in forensic cases involving various types of violence and sexual assault.

## Materials and methods

### Study material and ethics

Our department performs clinical forensic examinations in four of Denmark’s twelve national police districts. Worldwide, clinical forensic examinations can be requested by law enforcement, public prosecutors, and judges to support investigations and legal proceedings through thorough evidence collection and analysis. In Denmark, law stipulates that the police determine the necessity of an examination and request both forensic and toxicological analyses based on case relevance. In 2019, the department conducted 820 clinical forensic examinations, of which 558 involved adults over 18 years of age. Of these, 455 individuals provided urine samples, which were stored at -20 °C before analysis. These 455 urine samples constitute the material described in this study. The samples include individuals classified as alleged victims and alleged perpetrators, as their conviction status is unknown. For clarity, these individuals will be referred to as victims and perpetrators in the following sections. In addition to the biological samples, case documents, which include details about the individual implicated in the crime and the chronological events surrounding the case, were examined. Information such as gender, age, and the individual’s role in the case was recorded in a database. Subsequently, both the information and samples were anonymized to comply with national ethical guidelines for handling biological samples. Since the sample material was fully anonymized, ethical approval was not required under Danish guidelines.

### Analysis of urine samples

The 455 urine samples were analyzed using three different analytical methods. The substances tested were selected based on internal statistics identifying the most frequently detected drugs in national cases. First, ethanol concentration was determined using headspace gas chromatography with flame ionization, a well-established method described elsewhere [22]. Subsequently, the samples were analyzed for substances of abuse and medicinal compounds using two liquid chromatography-mass spectrometry (LC-MS) methods. These methods included a newly developed LC-MS/MS technique analyzing 62 compounds and a previously established UPLC-HR-qTOF-MS method, commonly used for toxicological screening [21]. Detailed methodology, sample preparation, and data processing are described in Supplementary Document 1. Validation results for the LC-MS/MS method are provided in Supplementary Document 2.

## Results and discussion

### Individuals presented in clinical forensic cases in 2019

On a national level, the police can request a clinical forensic examination for victims and perpetrators involved in criminal acts. These examinations assist in investigating the case by securing DNA samples, documenting injuries, and obtaining urine and blood samples for toxicological analysis. Clinical examinations are conducted only in cases where the police deem the results critical for the investigation; consequently, they are not performed in all criminal cases or for all individuals implicated. Typically, clinical examinations are reserved for more serious cases.

In 2019, 358 cases involving a total of 455 individuals over the age of 18 included urine sampling. These cases were categorized into six types of crime, as detailed in Table 1.

**Table 1:** Overview of the case types and individuals included in the study. The case type “Other” includes robbery, road traffic accidents, and suspicious homicide among others. The mean age of individuals for each category is presented in cases of more than one person and the min, max, and median values are presented for the categories with more than three persons to maintain the anonymization

Type of case	Role in case	Gender	Count	Age (mean)	Min-max (median)
**Blunt force** (*n* = 82)	Victim	Female	20	35	20–52 (32)
		Male	21	41	18–83 (34)
	Perpetrator	Female	4	45	39–53(45)
		Male	37	34	18–63 (31)
**Sharp force** (*n* = 73)	Victim	Female	3	46	-
		Male	25	31	19–61 (29)
	Perpetrator	Female	6	37	24–54(35)
		Male	39	31	18–57 (26)
**Shooting** (*n* = 7)	Victim	Female	-	-	-
		Male	5	29	20–36(31)
	Perpetrator	Female	-	-	-
		Male	2	38	-
**Pyromania** (*n* = 27)	Victim	Female	-	-	-
		Male	-	-	-
	Perpetrator	Female	2	51	-
		Male	25	31	18–67 (27)
**Sexual assault** (*n* = 254)	Victim	Female	174	26	18–68 (23)
		Male	3	22	-
	Perpetrator	Female	1	-	-
		Male	76	29	18–60 (26)
**Other** (*n* = 12)	Victim	Female	3	26	18–31(28)
		Male	2	32	-
	Perpetrator	Female	2	26	-
		Male	5	38	22–62 (28)
**Total** (*n* = 455)	Victim	Female	200	27	18–68(24)
		Male	56	34	18–83(29)
	Perpetrator	Female	15	40	24–60 (41)
		Male	184	31	18–67 (27)

As shown in Table 1, the most frequent case type was sexual assault, followed by blunt force and sharp force cases. Since 2016, there has been an increase in the number of victims and perpetrators examined in sexual assault cases, likely due to the Ministry of Justice’s directive for more thorough investigations in this area [23]. The remaining categories (pyromania, shooting, and others) were less common in the study. The average age of individuals varies across case types, but the majority fall within the 26–35 year age range. In 73 of the 358 cases, both a victim and a perpetrator were involved. Additionally, in nine cases, there were two to five perpetrators associated with a single victim.

#### Alcohol and substances detected in clinical forensic cases

Results from the analysis of 455 urine samples from clinical forensic examinations of adult victims and perpetrators are summerized in Table 2. For a comprehensive list of the compounds detected in each class, please refer to Supplementary Table 1.

**Table 2:** Prevalence of alcohol, drugs, medicals, and performance and image-enhancing drugs in the six categories of clinical forensic case types in the study distributed among victims (V) and perpetrators (P). Both percentages and the number of positive findings are listed in the table

	Blunt force		Sharp force		Sexual assault		Pyromania		Shooting		Other		Total	
	V	P	V	P	V	P	V	P	V	P	V	P	V	P
**Alcohol**	**29%** 12	**44%** 18	**36%** 10	**42%** 19	**35%** 62	**38%** 29	- -	**33%** 9	**40%** 2	**100%** 2	**20%** 1	**29%** 2	**34%** 87	**40%** 79
**Cannabis (THC**,** THC-COOH)**	**34%** 14	**44%** 18	**36%** 10	**51%** 23	**8%** 14	**10%** 8	**-** -	**19%** 5	**20%** 1	**-** -	**-** -	**57%** 4	**15%** 39	**29%** 58
**CNS stimulants**	**34%**	**39%**	**43%**	**40%**	**14%**	**15**	**-**	**19%**	**20%**	**100%**	**60%**	**29%**	**21%**	**28%**
*Amphetamine*	5	10	3	5	6	4	-	-	1	-	-	-	15	19
*Metamphetamine* ^*1*^ *and amphetamine*	1	1	-	1	1	3	-	2	-	-	-	-	2	7
*Methylphenidat* ^*1*^ *and ritalinic acid*	2	3	2	4	6	2	-	2	-	-	1	2	11	13
*MDMA*	-	1	1	3	4	3	-	1	-	-	-	-	5	8
*MDA*	-	1	-	3	1	-	-	-	-	-	-	-	1	4
*Cocaine and benzoylecgonine*	14	19	14	17	16	15	-	5	3	2	2	1	49	59
*Cocaethylene*	-	5	4	8	7	4	-	-	1	2	1	-	13	19
*Ephedrin*	1	-	1	-	-	-	-	-	-	-	-	-	2	-
**Opioids**	**32%**	**20%**	**50%**	**33%**	**6%**	**9**	**-**	**15%**	**80%**	**-**	**40%**	**29%**	**17%**	**18%**
**Benzodiazepines**	**24%**	**20%**	**4%**	**20%**	**7%**	**3**	**-**	**7%**	**20%**	**-**	**20%**	-	**10%**	**11%**
**Antipsychotic agents**	**20%**	**12%**	**11%**	**13%**	**7%**	**3**	**-**	**15%**	**-**	**-**	**-**	**29%**	**9%**	**10%**
**Anti-depressants**	**5%**	**5%**	**7%**	**4%**	**12%**	**8**	**-**	**15%**	**-**	**-**	**20%**	**14%**	**10%**	**8%**
**Performance and image-enhancing-related drugs**	**2%**	**10%**	**4%**	**7%**	**2%**	**5**	**-**	**11%**	**20%**	**-**	**-**	**-**	**3%**	**7%**
*Steroids T/E ratio > 6*	-	1	-	3	-	-	-	1	1	-	-	-	1	5
*Nandrolone and its meabolites*	-	1	-	2	-	-	-	1	-	-	-	-	-	4
*Boldenone and its metabolites*	-	-	-	2	-	-	-	-	-	-	-	-	-	2
*Trenbolone and its metabolites*	-	-	-	1	-	-	-	-	-	-	-	-	-	1
*Methenolone*	-	-	-	-	-	-	-	1	-	-	-	-	-	1
*Sildenafil*	1	4	-	-	-	4	-	-	-	-	-	-	1	8
*Anastrozole*	-	-	-	-	-	-	-	-	1	-	-	-	1	-
*Salbutamol*	-	-	1	1	4	-	-	1	-	-	-	-	5	2
**Lifestyle substances**	**93%**	**83%**	**89%**	**84%**	**73%**	**75**	**-**	**85%**	**60%**	**50%**	**80%**	**14%**	**78%**	**78%**
*Nicotine and cotinine*	33	30	19	34	103	53	-	18	1	1	3	-	159	136
*Paracetamol*	19	10	20	17	47	16	-	11	2	-	1	1	89	55
*Ibuprofen* ^*1*^	5	-	4	2	8	-	-	2	-	-	-	-	17	4
**Other**	**12%**	**5%**	**15%**	**2%**	**3%**	**-**	**-**	**4%**	**20%**	**-**	**20%**	**-**	**6%**	**2%**
No drugs and alcohol*	**24%** 10	**10%** 4	**4%** 1	**13%** 6	**40%** 71	**36%** 28	**-** -	**33%** **9**	**-** -	**-** -	**20%** 1	**14%** 1	**32%** 83	**19%** 48
No drugs and alcohol in cases ≤ 24 h*	**24%** 10	**5%** 2	**4%** 1	**13%** 6	**17%** 30	**22%** 17	**-**	**30%** 8	**-**	**-**	**20%** 1	**14%** 1	**16%** 42	**17%** 34
No drugs and alcohol in cases between 24 and 48 h*	**-**	**-**	**-**	**-**	**10%** 17	**13%** 10	**-**	**-**	**-**	**-**	**-**	**-**	**7%** 17	**5%** 10

* In this category, urine samples were allowed to contain nicotine, paracetamol, and ibuprofen without being classified as substances of abuse

^1^The compound is only included in the UPLC-HR-qTOF-MS screening method and therefore not verified by both analytical methods

Nearly all classes of substances were detected across the six case types (Table 2). Despite this, no substances were identified in 32% of victims and 19% of perpetrators. Alcohol was the most frequently detected compound, found in 34% of victims and 40% of perpetrators, excluding those in the lifestyle category. This finding is consistent with previous studies on both victims and perpetrators [6, 7]. CNS stimulants were detected in 21% of victims and 28% of perpetrators. Blunt and sharp force cases showed the highest percentage of CNS stimulants, a trend aligned with other studies [9, 10]. This is expected, as cocaine, the most commonly detected CNS stimulant, is known for its mood-altering effects and increased propensity for risky behavior, including violence [24]. Similarly, cannabis, which has also been linked to violent behavior [25], is frequently encountered in street-level drug reports by the Danish police [26]. [26].

The presence of opioids in blunt (32% in victims and 20% in perpetrators) and sharp force cases (50% in victims and 33% in perpetrators) is unexpected given their primary effects of analgesia and sedation. Despite their usual sedative effects, opioids have been associated with an increased risk of violence in various studies [27–29]. It should be noted that some opioids, such as fentanyl, might be detected due to medical treatment rather than self-administration. Steroids and other doping-related substances were found in approximately 2% of samples. A testosterone-to-epitestosterone (T/E) ratio between 4 and 6 was observed in 2% of urine samples, suggesting possible steroid use, though this was not confirmed by metabolites or other methods. This finding is consistent with a Danish study from 2014 [30], which also included cases involving driving under the influence. However, these figures are relatively low compared to a ten-year Swedish study, which found 33.5% of individuals testing positive for steroids [4].

When comparing drug occurrences between victims and perpetrators across all case types, a significant difference was observed (*p* < 0.01) in blunt and sharp force cases. However, there were no significant differences in sexual assault cases (*p* = 0.04). The higher number of detected substances in perpetrators compared to victims in blunt and sharp force cases may explain these differences. Conversely, more substances were detected in victims than in perpetrators in opioid cases. Due to small sample sizes, no tests were performed on other categories. No significant difference was found between perpetrators in blunt force and sharp force cases (*p* = 0.47), but significant differences were observed between perpetrators in blunt and sharp force cases versus those in sexual assault cases (*p* < 0.01). Interestingly, substances of abuse were detected in both victims and perpetrators in most case types, with some cases showing a higher frequency of substances in victims, such as CNS stimulants in sharp and blunt force cases. A possible explanation for this could be a higher incidence of criminal activities within drug-abusive environments.

Polydrug use is frequently observed among individuals engaged in criminal activities, which is crucial to recognize due to the escalated risks associated with simultaneous drug consumption. The number of substances detected in each case is summarized in Table 3.

**Table 3:** The number of substances detected in each case type. The occurrence of nicotine, Paracetamol, and ibuprofen was not included when counting the number of substances detected for each case type

Number of detected substances		1	2	3	4	5	> 5 (min-max value)
Blunt force	Victim	**17%** 7	**10%** 4	**17%** 7	**7%** 3	**10%** 4	**15%** 6 (*6–8*)
	Perpetrator	**15%** 6	**20%** 8	**22%** 9	**7%** 3	**10%** 4	**10%** 4 (*6–11*)
Sharp force	Victim	**25%** 7	**32%** 9	**7%** 2	**11%** 3	**14%** 4	**7%** 2 (*6–7*)
	Perpetrator	**22%** 10	**18%** 8	**13%** 6	**9%** 4	**7%** 3	**18%** 8 (*6–9*)
Sexual assault	Victim	**34%** 61	**14%** 25	**8%** 14	**1%** 2	**1%** 1	**3%** 5 (*6–8*)
	Perpetrator	**36%** 28	**12%** 9	**9%** 7	**3%** 2	**3%** 2	**1%** 1 (*7*)
Pyromania	Victim	-	-	-	-	-	-
	Perpetrator	**22%** 6	**26%** 7	**7%** 2	**7%** 2	-	**7%** 2 (*7–8*)
Shooting	Victim	**20%** 1	-	**40%** 2	**20%** 1	-	**20%** 1 (*7*)
	Perpetrator	-	**100%** 2	-	-	-	-
Other	Victim	-	**40%** 2	**20%** 1	-	-	**20%** 1 (*7*)
	Perpetrator	**43%** 3	-	**29%** 2	-	-	**14%** 1 (*6*)
Total	Victim	**30%** 76	**16%** 40	**10%** 26	**4%** 9	**4%** 9	**6%** 15 (*6–8*)
	Perpetrator	**27%** 53	**17%** 34	**13%** 26	**6%** 11	**5%** 9	**8%** 16 (*6–11*)

The range of drugs used in sharp force and blunt force cases varied from one to eleven, without a distinct pattern. This finding aligns with the known correlation between polydrug use and increased physical and verbal aggression [31]. In contrast, polydrug use in sexual assault cases was less common, with only a small percentage (5–7%) involving more than three substances among victims and perpetrators. This is consistent with other studies, which found either no drug use or only alcohol in sexual assault cases, with limited occurrences of polydrug use [13, 14, 16].

#### The interrelationship of drug patterns between victims and perpetrators

**Fig. 1:**
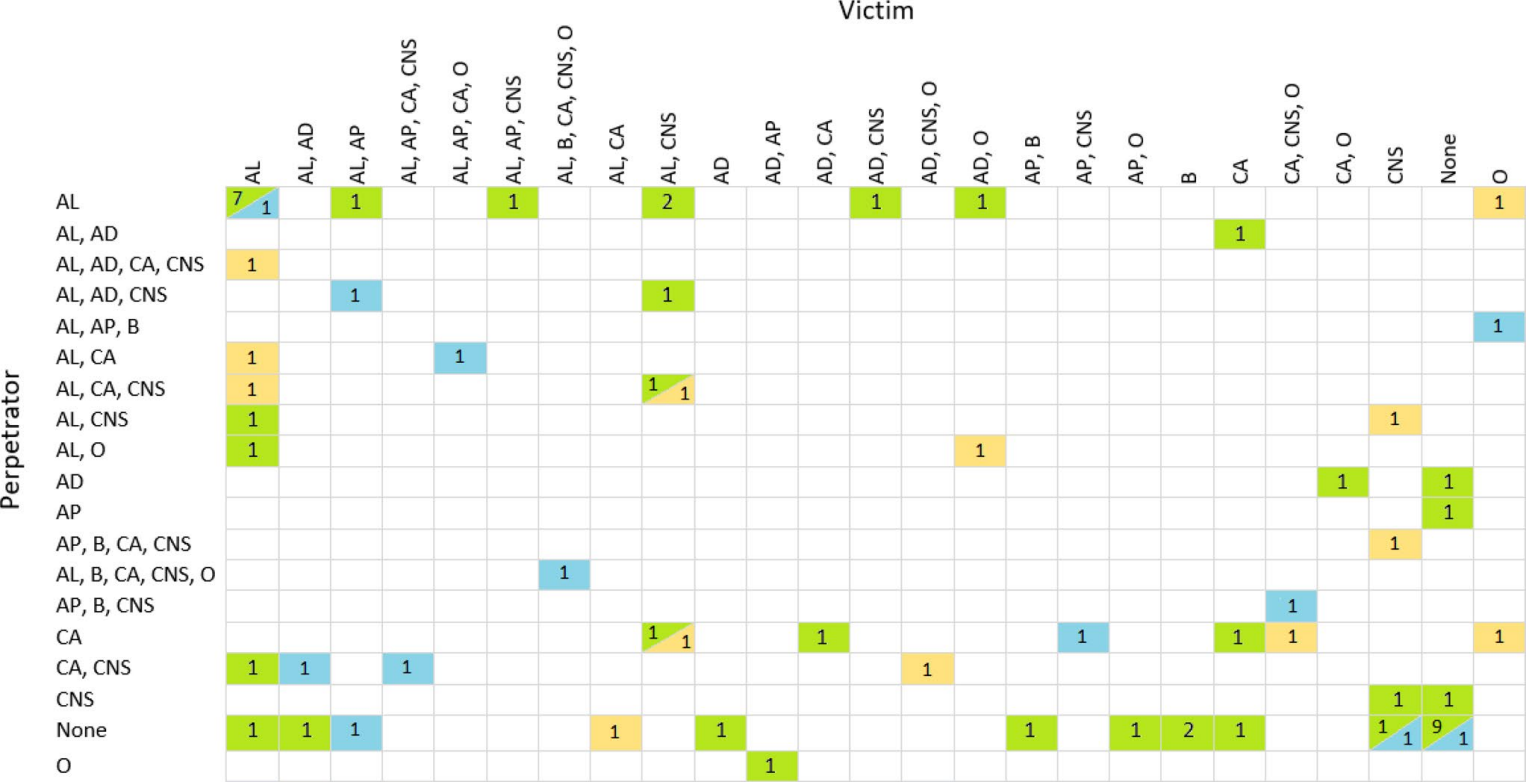
An overview of the interrelationship of alcohol and drug abuse between victim and perpetrator in cases where there is only one victim and one perpetrator. The illustration comprises 71 relationships, with 13 involving sharp force (blue), 12 involving blunt force (yellow), and the remaining 46 falling into the category of sexual assault (green). Abbreviation used in this Figure: AL: alcohol, AD: antidepressants, AP: antipsychotic agents, B: benzodiazepines, CA: cannabis, CNS: central nervous system stimulants, O: opioids

Understanding whether both the victim and the perpetrator were under the influence during the incident is crucial for accurately assessing the circumstances and the potential impact of substance use on the event. Figure 1 illustrates the relationship in 71 cases involving a single victim and a single perpetrator.

As shown in Fig. 1, both the victim and the perpetrator were under the influence of at least one substance in cases of sharp force and blunt force where both individuals were present. Most cases involving sexual assault exhibited a similar pattern between victims and perpetrators. However, in nine cases, no substances of abuse were detected in either the victim or the perpetrator. This unexpected finding contradicts the common assumption that victims use fewer drugs compared to perpetrators. It has been reported that perpetrators often come from environments characterized by high levels of polydrug use, which may increase their likelihood of engaging in criminal activities [1, 3].

The sampling interval for victims ranged from one to 24 h, while for perpetrators it ranged from one to eight hours, except for one case with a 40-hour interval, suggesting that late sampling could contribute to negative results. Additionally, there were nine cases with multiple perpetrators for each victim. Details of these cases are provided in Supplementary Table 2.

Currently, toxicological analyses are requested by the police in less than 10% of clinical forensic cases. This limited analysis provides only a partial understanding of this population segment. Gaining insight into the types and quantities of substances used, as well as the combinations and associated harms, is crucial for developing effective information campaigns and prevention strategies aimed at reducing criminal incidents. Regular toxicological analyses could also help monitor the effectiveness of various interventions in this area.

#### The importance of the timing of sample collection for results in clinical forensic cases

**Fig. 2:**
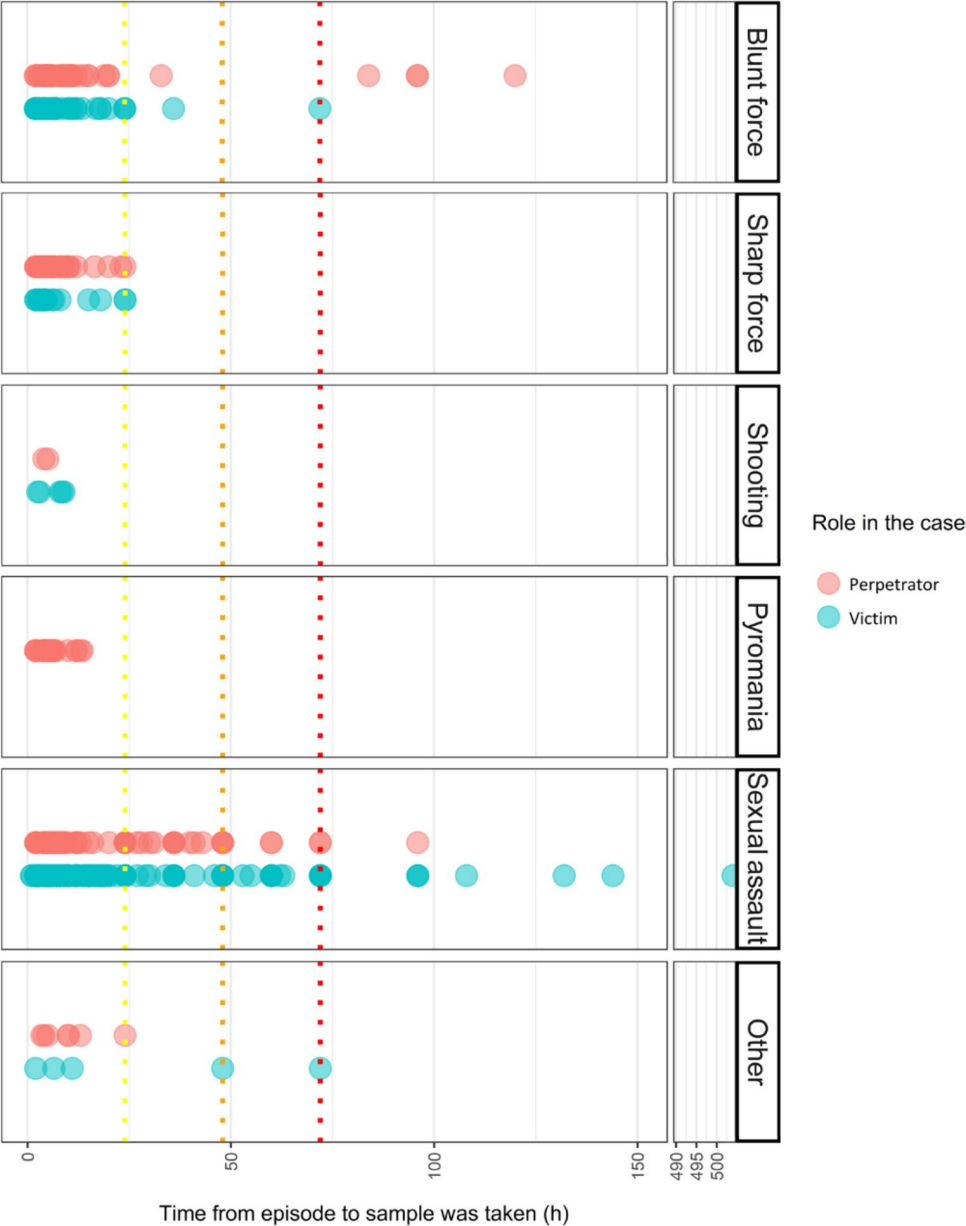
The time from the incident until the sample was taken by a forensic pathologist. The time distribution was 367 samples ≤ 24 h (yellow line) < 44 samples ≤ 48 h (orange line) < 28 samples ≤ 72 h (red line), 12 > 72 h. The time interval was not registered for four samples

For optimal accuracy, urine samples should be collected as soon as possible after an incident. Delays in sampling increase the risk of contamination and degradation of drug metabolites, which complicates the determination of substances present and their concentrations. This compromises the reliability of forensic toxicology results in determining whether an individual was under the influence at the time of the crime. Figure 2 illustrates the time interval from the incident to sample collection.

Figure 2 shows that 367 samples, representing 81% of the total, were collected within 24 h of the incident. 10% were collected between 24 and 48 h, 6% between 48 and 72 h, and 3% after 72 h. Most late samplings involved alleged victims in sexual assault cases, with some perpetrators in blunt force cases also falling into this category, possibly due to delayed arrests. Only 1% of the samples lacked a defined time interval. This unknown interval often resulted from inability to recall the timing of the incident or post-registration for cases reported over an extended period.

The detection window for most illegal drugs in urine is approximately 48 h, though some can be detected for longer after a single dose [32]. For individuals with daily drug consumption, the detection window may extend to several days or even months, depending on the drug’s administration and other factors [32]. Thus, the fact that most urine samples were collected within 24 to 48 h enhances the reliability of the results.

**Fig. 3:**
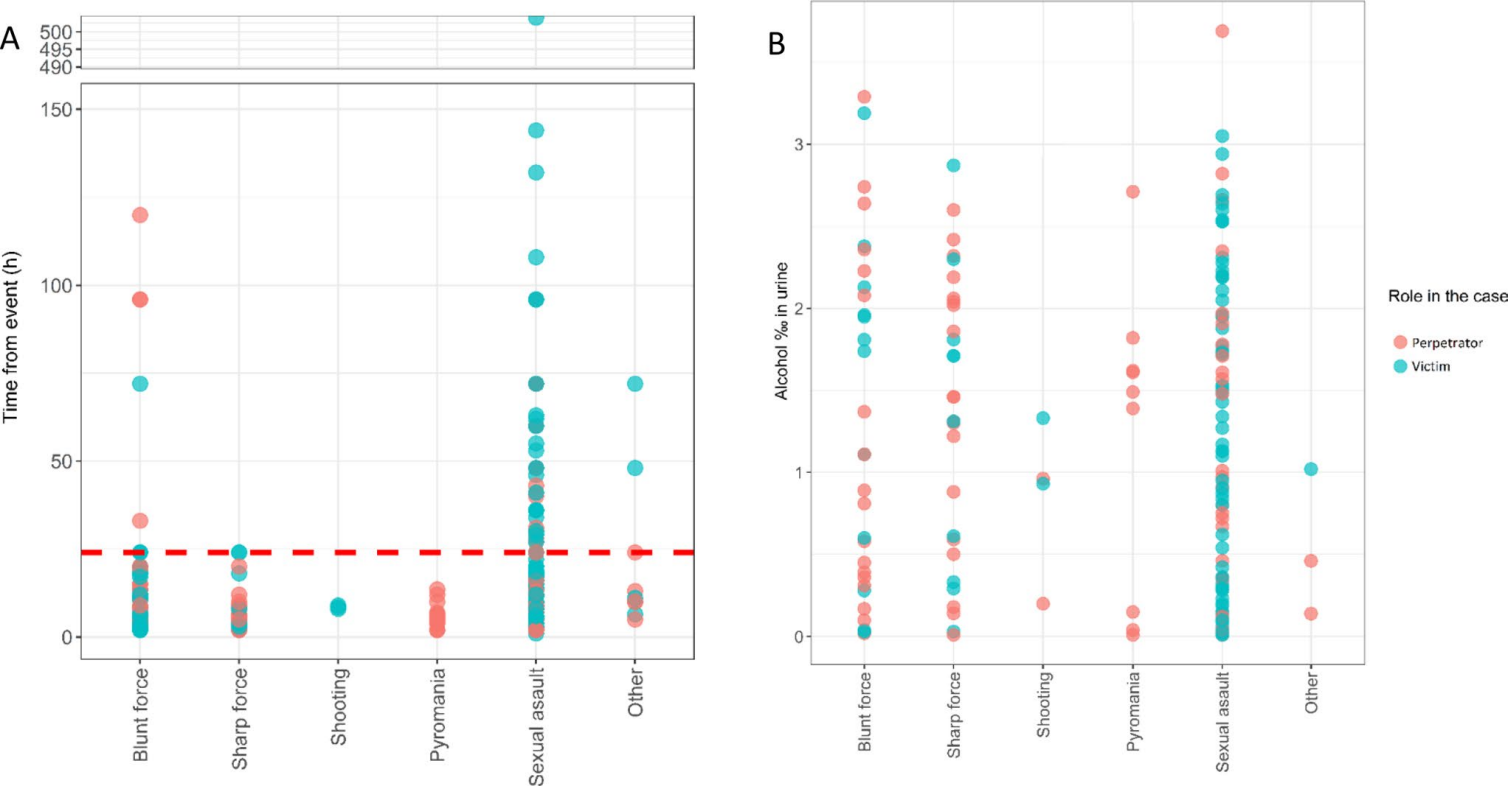
**A**) An overview of the timeline for cases where alcohol was not detected. For 209 out of the 286 negative sample, the time interval from the incident to sampling was found below 24 h and for the remaining 77 samples, the time interval was above 24 h. **B**) An overview of the alcohol level in urine for 169 samples with a confirmed positive detection

Alcohol has a shorter detection window in urine compared to most substances. Detection time can range from approximately one to 24 h, influenced by factors such as intake amount, diet, and genetics. To assess whether the absence of alcohol in urine samples was due to delayed sampling or a true negative result, Fig. 3A shows the collection times for negative alcohol samples. Additionally, Fig. 3B depicts the alcohol levels in urine samples across different case types.

As seen in Figs. 3A and 209 corresponding to 73% of the negative results were from samples collected within 24 h. Thus, it is reasonable to conclude that these samples were negative for alcohol at the time of the incident. For the remaining 27% of negative results, delayed sampling could mean that individuals might have been under the influence of alcohol during the crime, but this cannot be conclusively determined.

Alcohol-positive findings in the urine ranged from 0.01‰ to 3.65‰, as seen in Fig. 3B. Positive results included both low and high levels of alcohol in victims and perpetrators across blunt force, sharp force, and sexual assault cases. I should be mentioned that the urine-to-blood alcohol ratio averages 1.3, but can vary between 1.0 and 2.0 depending on whether the urine is in the absorptive or post-absorptive phase [33].

### Strengths and limitations of the study

Most published studies have concentrated on either the victim, the perpetrator, or a combination of both categories within a single group. The main advantage of this study is its ability to examine the substances consumed by both the victim and the perpetrator across different case types, and to investigate the correlation between these substances when both are present. Additionally, the use of two analytical methods for sample analysis of substance use enhances the robustness of the results. However, not all detected compounds were included in the targeted LC-MS/MS method, resulting in some compounds being detected by only one analytical method. The substances selected for analysis were based on national statistical data and practical considerations aimed at streamlining processing and conserving time. While some relevant substances might be missing, the TOF-MS data collected enables retrospective reprocessing to identify additional compounds, potentially offering a more comprehensive overview of substance use within our sample groups.

Regarding limitations, 153 individuals in our sample lacked a corresponding case registration. These non-linked victims and perpetrators may not be included in our sample due to being under 18 years of age, a forensic clinical examination not being performed or being performed at a different forensic department, the inability of the police to trace the perpetrator, or the absence of a urine sample. Additionally, in a few cases, victims declined to undergo examination, resulting in the absence of sample collection.

A clinical forensic examination should ideally occur as close to the time of the crime as possible to strengthen the findings. As discussed in Section. “Alcohol and substances detected in clinical forensic cases”, some cases were reported or discovered by the police later than 48 h post-incident. Knowing the time interval between the criminal event and the biological sample collection is crucial for interpreting results. Detecting drugs and their metabolites in urine becomes increasingly difficult if the sample is collected after the detection window for the drug of interest has closed. When the time interval surpasses the detection window of a specific drug, a negative result might be attributed to delayed sampling rather than the absence of drug consumption. Moreover, we cannot determine if some drugs were ingested before the incident or during the interval between the incident and urine sample collection. Lastly, as noted in Section. “Study material and ethics”, individuals undergoing clinical forensic examinations are selected and referred by the Danish police, which means that not all individuals involved in various types of crimes are represented in our sample. Therefore, it is unclear whether our sample is representative of all victims and perpetrators in Denmark. Furthermore, the selection process for clinical forensic examinations may differ across countries and authorities, warranting caution when comparing studies based on individuals who have undergone such examinations.

## Conclusion

The analysis of 455 clinical forensic examinations from 2019 provided valuable insights into alcohol and drug abuse among victims and perpetrators. The study identified substances of abuse in the majority of both victims and perpetrators across various types of violence and sexual assault. Alcohol was the most frequently detected substance across all case types. Cannabis and central nervous system (CNS) stimulants were also frequently found, consistent with patterns observed in street-level drug seizures by Danish police. Other substances detected included opioids, benzodiazepines, antidepressants, antipsychotic drugs, and steroids.

Post-hoc tests revealed a significant difference in drug use between victims and perpetrators in blunt force and sharp force cases. No significant difference was observed in sexual assault cases. In sexual assault cases, both victims and perpetrators were often not under the influence of substances; when a substance was detected in perpetrators, the same type of substance was frequently found in related victims. Additionally, substances were often detected more frequently in victims than in perpetrators.

Overall, this study offers important information about the prevalence of alcohol and drug abuse among individuals involved in forensic examinations. The forensic analysis of these patterns, combined with insights from psychology and other relevant disciplines, can contribute significantly to understanding the impact of substances on crime commission and their effects on both victims and perpetrators. This information is crucial for developing targeted interventions aimed at reducing future criminal activities.

## Key points

1. Alcohol was the most frequently detected substance of abuse.
2. No drugs or alcohol were found in 32% of victims and 19% of perpetrators overall.
3. In each crime type, victims and offenders exhibited comparable substance use.
4. Polydrug and overall drug use were more frequent in violent cases than in sexual assaults.

## Electronic supplementary material

Below is the link to the electronic supplementary material.

Supplementary Material 1

Supplementary Material 2

Supplementary Material 3

## Data Availability

The data that support the findings of this study are available from the corresponding author, upon reasonable request.
