# Supplementary material for: Detection of substance use in clinical forensic cases: urine analysis of victims and perpetrators
Source: Forensic Sci Med Pathol. 2024 Sep 5;21(2):522–31. doi: 10.1007/s12024-024-00873-w (PMC12325547; doi:10.1007/s12024-024-00873-w)
Supplement: Supplementary file 1 — Supplementary Material 1 [file 12024_2024_873_MOESM1_ESM.docx]

Supplementary Tables

**Supplementary Table 2:** The full table of the occurrence of alcohol, narcotic drugs, medicals, and performance and image-enhancing drugs in the six categories of clinical forensic case types in the study. The percentages shown for each category relate to the number of individuals where the compound class of interest has been detected in.

|  | **Blunt force** | | **Sharp force** | | **Sexual assault** | | **Pyromania** | | **Shooting** | | **Other** | | **Total** | |
| --- | --- | --- | --- | --- | --- | --- | --- | --- | --- | --- | --- | --- | --- | --- |
|  | V | P | V | P | V | P | V | P | V | P | V | P | V | P |
| **Alcohol** | **29%**  12 | **44%**  18 | **36%**  10 | **42%**  19 | **35%**  62 | **38%**  29 | -  - | **33%**  9 | **40%**  2 | **100%**  2 | **20%**  1 | **29%**  2 | **34%**  87 | **40%**  79 |
| **Cannabis (THC, THC-COOH)** | **34%**  14 | **44%**  18 | **36%**  10 | **51%**  23 | **8%**  14 | **10%**  8 | **-**  - | **19%**  5 | **20%**  1 | **-**  - | **-**  - | **57%**  4 | **15%**  39 | **29%**  58 |
| **CNS stimulants** | **34%** | **39%** | **43%** | **40%** | **14%** | **15%** | **-** | **19%** | **20%** | **100%** | **60%** | **29%** | **21%** | **28%** |
| *Amphetamine* | 5 | 10 | 3 | 5 | 6 | 4 | - | 0 | 1 | - | - | - | 15 | 19 |
| *Methamphetamine^1^ and amphetamine* | 1 | 1 | 0 | 1 | 1 | 3 | - | 2 | - | - | - | - | 2 | 7 |
| *Methylphenidat^1^ and ritalinic acid* | 2 | 3 | 2 | 4 | 6 | 2 | - | 2 | - | - | 1 | 2 | 11 | 13 |
| *MDMA* | - | 1 | 1 | 3 | 4 | 3 | - | 1 | - | - | - | - | 5 | 8 |
| *MDA* | - | 1 | 0 | 3 | 1 | 0 | - | 0 | - | - | - | - | 1 | 4 |
| *Cocaine and benzoylecgonine* | 14 | 19 | 14 | 17 | 16 | 15 | - | 5 | 3 | 2 | 2 | 1 | 49 | 59 |
| *Cocaethylene* | 0 | 5 | 4 | 8 | 7 | 4 | - | - | 1 | 2 | 1 | - | 13 | 19 |
| *Ephedrin* | 1 | - | 1 | - | - | - | - | - | - | - | - | - | 2 | 0 |
| **Opioids** | **32%** | **20%** | **50%** | **33%** | **6%** | **9%** | **-** | **15%** | **80%** | **-** | **40%** | **29%** | **17%** | **18%** |
| *Heroin (6-MAM^1^and morphine^2^)* | 1 | 1 | - | 1 | - | - | - | - | - | - | - | - | 1 | 2 |
| *Morphine* | 8 | 18 | 5 | 8 | 9 | 5 | - | 3 | 2 | - | - | 1 | 24 | 35 |
| *Methadone and EDDP* | 7 | 4 | 1 | 11 | 1 | - | - | 2 | 1 | - | 1 | 1 | 11 | 18 |
| *Fentanyl^1^* | 3 | - | 6 | - | - | - | - | - | - | - | - | - | 9 | 0 |
| *Oxycodon^1^* | 1 | 1 | 1 | - | 1 | - | - | - | - | - | - | - | 3 | 1 |
| *Tramadol^1^* | 2 | 2 | 6 | 1 | 3 | 2 | - | 1 | 2 | - | 1 | - | 14 | 6 |
| *Codein^1^* | 3 | 2 | - | 3 | - | - | - | - | - | - | - | - | 3 | 5 |
| **Benzodiazepines** | **24%** | **20%** | **4%** | **20%** | **7%** | **3%** | **-** | **7%** | **20%** | **-** | **20%** | - | **10%** | **11%** |
| *Diazepam* | 2 | 3 | - | 6 | 2 | 1 | - | 1 | - | - | 1 | - | 5 | 11 |
| *Clonazepam^1^* | 7 | 4 | - | 3 | 1 | 1 | - | - | - | - | 1 | - | 9 | 8 |
| *Zopiclon^1^* | - | - | - | 1 | 2 | - | - | - | - | - | - | - | 2 | 1 |
| *Zolpidem^1^* | - | - | - | - | 1 | - | - | - | - | - | - | - | 1 | 0 |
| *Nitrazepam^1^* | 2 | - | - | 1 | - | - | - | - | - | - | - | - | 2 | 1 |
| *Triazolam^1^ and triazolam-α-OH^1^* | - | - | - | - | - | - | - | 1 | - | - | - | - | 0 | 1 |
| *Chlordiazepoxid^1^* | - | - | 1 | - | 2 | - | - | - | - | - | - | - | 3 | 0 |
| *Oxazepam^1^* | 3 | 3 | - | 7 | 8 | 1 | - | 1 | 1 | - | 1 | - | 13 | 12 |
| *Alprazolam^1^ and Alprazolam-α-OH^1^* | 3 | 1 | - | - | 1 | - | - | - | 1 | - | 1 | - | 6 | 1 |
| **Antipsychotic agents** | **20%** | **12%** | **11%** | **13%** | **7%** | **3%** | **-** | **15%** | **-** | **-** | **-** | **29%** | **9%** | **10%** |
| *Citalopam and escitalopam^1^* | 3 | 2 | 1 | 2 | 4 | - | - | 1 | - | - | - | - | 8 | 5 |
| *Qutiapine^1^* | 6 | 3 | 2 | 4 | 5 | - | - | 2 | - | - | - | - | 13 | 9 |
| *Chlorprothixen^1^* | - | - | - | - | - | - | - | 1 | - | - | - | 1 | 0 | 2 |
| *Amisulprid^1^* | - | - | - | - | 1 | 1 | - | - | - | - | - | - | 1 | 1 |
| *Olanzapin^1^* | - | - | - | - | 2 | 1 | - | - | - | - | - | - | 2 | 1 |
| *Aripiprazol^1^* | - | - | - | - | 4 | - | - | - | - | - | - | 1 | 4 | 1 |
| **Anti-depressants** | **5%** | **5%** | **7%** | **4%** | **12%** | **8%** | **-** | **15%** | **-** | **-** | **20%** | **14%** | **10%** | **8%** |
| *Fluoxetin ^1^* | 2 | - | - | 2 | 9 | 5 | - | 1 | - | - | - | - | 11 | 8 |
| *Sertralin^1^* | 2 | 1 | 1 | - | 13 | 1 | - | 3 | - | - | 1 | 1 | 17 | 6 |
| *Paroxetin^1^* | - | - | 1 | - | 1 | - | - | - | - | - | - | - | 2 | 0 |
| **Performance and image enhancing drugs** | **2%** | **10%** | **4%** | **7%** | **2%** | **5%** | **-** | **11%** | **20%** | **-** | **-** | **-** | **3%** | **7%** |
| *Steroids T/E ratio >6* | - | 1 | - | 3 | - | - | - | 1 | 1 | - | - | - | 1 | 5 |
| *Nandrolone and its meabolites* | - | 1 | - | 2 | - | - | - | 1 | - | - | - | - | - | 4 |
| *Boldenone and its metabolites* | - | - | - | 2 | - | - | - | - | - | - | - | - | - | 2 |
| *Trenbolone and its metabolites* | - | - | - | 1 | - | - | - | - | - | - | - | - | - | 1 |
| *Methenolone* | - | - | - | - | - | - | - | 1 | - | - | - | - | - | 1 |
| *Sildenafil* | 1 | 4 | - | - | - | 4 | - | - | - | - | - | - | 1 | 8 |
| *Anastrozole* | - | - | - | - | - | - | - | - | 1 | - | - | - | 1 | - |
| *Salbutamol* | - | - | 1 | 1 | 4 | - | - | 1 | - | - | - | - | 5 | 2 |
| **Lifestyle substances** | **93%** | **83%** | **89%** | **84%** | **73%** | **75%** | **-** | **85%** | **60%** | **50%** | **80%** | **14%** | **78%** | **78%** |
| *Nicotine and cotinine* | 33 | 30 | 19 | 34 | 103 | 53 | - | 18 | 1 | 1 | 3 | - | 159 | 136 |
| *Paracetamol* | 19 | 10 | 20 | 17 | 47 | 16 | - | 11 | 2 | - | 1 | 1 | 89 | 55 |
| *Ibuprofen^1^* | 5 | - | 4 | 2 | 8 | - | - | 2 | - | - | - | - | 17 | 4 |
| **Other** | **12%** | **5%** | **15%** | **2%** | **3%** | **-** | **-** | **4%** | **20%** | **-** | **20%** | **-** | **6%** | **2%** |
| *Pregabalin^1^* | 2 | 2 | 1 | - | 4 | - | - | - | - | - | 1 | - | 8 | 2 |
| *Ketamine and Norketamine^1^* | 3 | - | 3 | 1 | 1 | - | - | 1 | 1 | - | - | - | 8 | 2 |
| **No drugs and alcohol*** | **24%**  10 | **10%**  4 | **4%**  1 | **13%**  6 | **40%**  71 | **36%**  28 | **-**  - | **33%**  **9** | **-**  - | **-**  - | **20%**  1 | **14%**  1 | **32%**  83 | **19%**  48 |
| **No drugs and alcohol in cases ≤ 24 h*** | **24%**  10 | **5%**  2 | **4%**  1 | **13%**  6 | **17%**  30 | **22%**  17 | **-** | **30%**  8 | **-** | **-** | **20%**  1 | **14%**  1 | **16%**  42 | **17%**  34 |
| **No drugs and alcohol in cases between 24 and 48 h*** | **-** | **-** | **-** | **-** | **10%**  17 | **13%**  10 | **-** | **-** | **-** | **-** | **-** | **-** | **7%**  17 | **5%**  10 |

* In this category, urine samples were allowed to contain nicotine, paracetamol, and ibuprofen without being classified as substances of abuse.

^1^The compound is only included in the UPLC-HR-qTOF-MS screening method and therefore not verified by both analytical methods.

**Supplementary Table 2**: An overview of the results from cases where there was one victim and more than one perpetrator.

|  | Victim | Perpetrator | | | | |
| --- | --- | --- | --- | --- | --- | --- |
|  |  | 1 | 2 | 3 | 4 | 5 |
| Shooting | Alcohol and CNS | Alcohol and CNS | Alcohol and CNS | - | - | - |
|  | Antipsychotic agents, cannabis, CNS, and opioid | Nothing (sampling interval is 2h) | Alcohol, cannabis, CNS, and opioid | - | - | - |
|  | Nothing (sampling interval is 3h) | Alcohol, cannabis, benzodiazepine, CNS, and opioid | Alcohol, cannabis, benzodiazepine, CNS, and opioid | Alcohol, antipsychotic agent, benzodiazepine, CNS, and opioid | Benzodiazepine, cannabis, CNS, and opioid | Alcohol, antipsychotic agent, benzodiazepine, and CNS |
| Bunt force | Alcohol, benzodiazepine, and cannabis | Benzodiazepine, cannabis, CNS, and opioid | Alcohol, antipsychotic agent, benzodiazepine, cannabis, and CNS | - | - | - |
|  | Alcohol and cannabis | Alcohol | Alcohol and antipsychotic agent | Alcohol, benzodiazepine, and cannabis | - | - |
|  | Alcohol and opioid | CNS | Cannabis | Cannabis | - | - |
|  | Antipsychotic agent, benzodiazepine, CNS and opioid | Benzodiazepine, CNS and opioid | Benzodiazepine, cannabis, CNS and opioid | - | - | - |
|  | Cannabis and CNS | CNS | Alcohol and CNS | Cannabis and CNS | - | - |
| Sexual assault | Nothing (sampling interval is 4h) | Nothing (sampling interval is 6h) | Antidepressive agent | - | - | - |
