# Supplementary material for: Detection of substance use in clinical forensic cases: urine analysis of victims and perpetrators
Source: Forensic Sci Med Pathol. 2024 Sep 5;21(2):522–31. doi: 10.1007/s12024-024-00873-w (PMC12325547; doi:10.1007/s12024-024-00873-w)
Supplement: Supplementary file 2 — Supplementary Material 2 [file 12024_2024_873_MOESM2_ESM.docx]

Supplementary document 1

Materials and methods specified

## *Chemicals and reagents*

All chemicals and solvents used were reagent grade or better. Methanol (MeOH), acetonitrile, formic acid, and ammonium acetate were purchased from Merck Life Science. β-glucuronidase from E. coli K12 (140 U/mg at 37 ^o^C, pH=7) was obtained from Roche Diagnostics GmbH (Mannheim, Germany). Ultra-pure water was obtained from a Merck Millipore Milli-Q^TM^ system (Merck LifeScience, Darmstadt, Germany ). The 62 compounds included in the LC-MS/MS method and their respective supplier are listed below in Table S1.1. The 62 selected compounds comprise the most commonly identified drugs of abuse in the routine toxicological screening of biological samples and a subset of doping agents. Stock solutions of each standard compound were prepared in MeOH at levels of 0.01 to 1 mg/mL and stored in ampoules at -20 °C. In addition, two stock solutions containing a mixture of all 62 compounds and the eight deuterated standards, respectively, were prepared in MeOH at a concentration of 0.01 mg/mL. Working solutions were prepared by diluting the stock solutions with MeOH to the desired concentrations.

**Table S1.1**: Optimized parameters for each compound included in the analytical method, and information of the compound and its supplier.

| **Compound** | **Specifications** | **Supplier** | **MRM trasitions (*m/z*)** | **DP**  **(V)** | **EP**  **(V)** | **CE**  **(V)** | **CXP**  **(V)** |
| --- | --- | --- | --- | --- | --- | --- | --- |
| 16β-hydroxystanozolol | Metabolite of stanozolol | NMI (North Ryde, NSW, Australia) | \| 345.2 🡪91 \| \| --- \| \| 345.2 🡪119.1 \| | 55 | 6 | 90 | 16 |
| 17-epioxandrolone | Metabolite of oxandrolone | NMI (North Ryde, NSW, Australia) | \| 307.1 🡪 289.1 \| \| --- \| \| 307.1 🡪 229.1 \| | 30 | 12 | 16 | 22 |
| 17-α-trenbolone | Metabolite of trenbolone | NMI (North Ryde, NSW, Australia) | \| 271.1 🡪 253.1 \| \| --- \| \| 271.1 🡪199.1 \| | 70 | 11 | 28 | 31 |
| 17α-methyl-5α-androstane-3α.17β-diol | Metabolite of methyltestosterone | NMI (North Ryde, NSW, Australia) | \| 271.3 🡪161.2 \| \| --- \| \| 271.3 🡪135.2 \| | 80 | 10 | 24 | 25 |
| 17α-methyl-5β-androstane-3α.17β-diol | Metabolite of methyltestosterone | NMI (North Ryde, NSW, Australia) | \| 271.3 🡪161.2 \| \| --- \| \| 271.3 🡪135.2 \| | 80 | 10 | 25 | 21 |
| 19-norandrosterone | Metabolite of nandrolone | NMI (North Ryde, NSW, Australia) | \| 277.1 🡪259.2 \| \| --- \| \| 277.1 🡪241.2 \| | 44 | 5 | 12 | 26 |
| 19-noretiocholanolone | Metabolite of nandrolone | NMI (North Ryde, NSW, Australia) | \| 277.2 🡪241.3 \| \| --- \| \| 277.2 🡪259.2 \| | 42 | 8.5 | 19 | 22 |
| 2α-methyl-5α-androstan-3α-ol-17-one | Metabolite of drostanolone | NMI (North Ryde, NSW, Australia) | \| 287.4 🡪269.4 \| \| --- \| \| 287.4 🡪145.3 \| | 136 | 10 | 17 | 17 |
| 1-Methylene-5α-androstan-3α-ol-17-one | Metabolite of methenolone | NMI (North Ryde, NSW, Australia) | \| 303.3 🡪285.4 \| \| --- \| \| 303.3 🡪267.3 \| | 157 | 12 | 15 | 14.5 |
| 3'-hydroxystanozolol | Metabolite of stanozolol | NMI (North Ryde, NSW, Australia) | \| 345.2 🡪97 \| \| --- \| \| 345.2 🡪 119 \| | 25 | 8 | 51 | 43 |
| 4-hydroxytamoxifen | Metabolite of tamoxifen | NMI (North Ryde, NSW, Australia) | \| 388.2 🡪222.7 \| \| --- \| \| 388.2 🡪315.9 \| | 85 | 11 | 31 | 35 |
| 5β-androst-1-en-17β-ol-3-one | Metabolite of boldenone | NMI (North Ryde, NSW, Australia) | \| 289.1 🡪271.2 \| \| --- \| \| 289.1 🡪252.9 \| | 98 | 11.5 | 19 | 31.5 |
| 5β-androst-1-en-3α-ol-17-one | Metabolite of boldenone | NMI (North Ryde, NSW, Australia) | \| 289.1 🡪253 \| \| --- \| \| 289.1 🡪145.1 \| | 21 | 5 | 16 | 29 |
| 9α-fluoro-17α-methyl-4-androsten-3α. 6β. 11β.17β-tetrol | Metabolite of fluoxymesterone | NMI (North Ryde, NSW, Australia) | \| 337.4 🡪299.1 \| \| --- \| \| 337.4 🡪317.2 \| | 125 | 10 | 19 | 20 |
| Amphetamine | CNS stimulant | Merck Life Science (St. Louis, MO, USA) | \| 136.0 🡪91.1 \| \| --- \| \| 136.0 🡪119.1 \| | 12 | 5 | 23 | 15 |
| Anastrozole | NSAI | SelleckChem (Houston, TX, USA) | \| 294.1 🡪 225.1 \| \| --- \| \| 294.1 🡪210.1 \| | 40 | 5 | 29 | 27 |
| Benzoylecgonine | Metabolite of cocaine | Merck Life Science (St. Louis, MO, USA) | \| 290.1 🡪168.2 \| \| --- \| \| 290.1 🡪105.4 \| | 25 | 5 | 25 | 21 |
| Boldenone | Steroid | Merck Life Science (St. Louis, MO, USA) | \| 287.2 🡪121 \| \| --- \| \| 287.2 🡪135.1 \| | 65 | 9 | 27 | 10 |
| Caffeine | Lifestyle drug and CNS stimulant | Merck Life Science (St. Louis, MO, USA) | 195.1 🡪137.9 | 53 | 9 | 28 | 21.5 |
| Citalopram | Antipsychotic agent | Merck Life Science (St. Louis, MO, USA) | \| 325.0 🡪234 \| \| --- \| \| 325.0 🡪247 \| | 80 | 10 | 38 | 20 |
| Clenbuterol | β-2 agonist | Merck Life Science (St. Louis, MO, USA) | \| 277.0 🡪203 \| \| --- \| \| 277.0 🡪132 \| | 40 | 8 | 22 | 10 |
| Clomiphene | SERM | Merck Life Science (St. Louis, MO, USA) | \| 406.1 🡪100.1 \| \| --- \| \| 406.1 🡪241.1 \| | 73 | 8 | 29.5 | 12 |
| Cocaethylene | Metabolite of alcohol and cocaine in a combination | Merck Life Science (St. Louis, MO, USA) | \| 318.0 🡪196.2 \| \| --- \| \| 318.0 🡪150.2 \| | 60 | 6 | 26 | 11 |
| Cocaine | CNS stimulant | Merck Life Science (St. Louis, MO, USA) | \| 304.3 🡪182.2 \| \| --- \| \| 304.3 🡪150.2 \| | 18 | 4.5 | 25.8 | 12 |
| Cotinine | Metabolite of nicotine | Merck Life Science (St. Louis, MO, USA) | \| 177.2 🡪80.1 \| \| --- \| \| 177.2 🡪98 \| | 47 | 7 | 35 | 18 |
| Creatinine | Endogenous alpha amino acid | Merck Life Science (St. Louis, MO, USA) | \| 114.0 🡪44 \| \| --- \| \| 114.0 🡪86.1 \| | 33 | 9 | 23 | 9 |
| Dehydroepiandrosterone | Endogenous steroid | NMI (North Ryde, NSW, Australia) | \| 289.1 🡪253.101 \| \| --- \| \| 289.1 🡪213.1 \| | 21 | 5 | 16 | 29 |
| Diazepam | Benzodiazepine | Merck Life Science (St. Louis, MO, USA) | \| 285.1 🡪193.1 \| \| --- \| \| 285.1 🡪154.1 \| | 40 | 6 | 45 | 13 |
| EDDP | Metabolite of methadone | Merck Life Science (St. Louis, MO, USA) | \| 278.1 🡪233.9 \| \| --- \| \| 278.1 🡪249.3 \| | 95 | 8 | 40 | 28 |
| Enobosarm (also known as ostarine) | SARM | SelleckChem (Houston, TX, USA) | \| 390.0 🡪193 \| \| --- \| \| 390.0 🡪120 \| | 60 | 5 | 42 | 19 |
| Ephedrine | CNS stimulant | Merck Life Science (St. Louis, MO, USA) | \| 166.4 🡪148.1 \| \| --- \| \| 166.4 🡪133 \| | 30 | 10 | 16 | 15 |
| Epitestosterone | Endogenous steroid | Cerilliant (Austin, TX, USA) | \| 289.4 🡪97.1 \| \| --- \| \| 289.4 🡪109.1 \| | 70 | 5 | 30 | 12 |
| Epitestosterone glucuronic acid | Phase II metabolite of epitestosterone | NMI (North Ryde, NSW, Australia) | \| 465.5 🡪97.1 \| \| --- \| \| 465.5 🡪109.1 \| | 100 | 5 | 38 | 11 |
| Epitestosterone sulphate | Phase II metabolite of epitestosterone | NMI (North Ryde, NSW, Australia) | \| 369.4 🡪97 \| \| --- \| \| 369.4 🡪109.1 \| | 135 | 6 | 32 | 12 |
| Flunitrazepam | AAS | NMI (North Ryde, NSW, Australia) | \| 314.1 🡪268.1 \| \| --- \| \| 314.1 🡪238.9 \| | 100 | 6 | 35 | 23 |
| Fluoxymesterone | AAS | NMI (North Ryde, NSW, Australia) | \| 337.3 🡪281.3 \| \| --- \| \| 337.3 🡪317.1 \| | 75 | 5 | 31 | 13 |
| Heroin | Opioid | Merck Life Science (St. Louis, MO, USA) | \| 370.2🡪268.2 \| \| --- \| \| 370.2 🡪328.1 \| | 62 | 11 | 39 | 26 |
| Letrozol | Aromatase inhibitor | Merck Life Science (St. Louis, MO, USA) | \| 286.0 🡪217.1 \| \| --- \| \| 286.0 🡪190 \| | 22 | 4 | 18 | 15 |
| Ligandrol | SARM | SelleckChem (Houston, TX, USA) | \| 339.1 🡪199 \| \| --- \| \| 339.1🡪220 \| | 143 | 8 | 41 | 19 |
| MDA | CNS stimulant | Merck Life Science (St. Louis, MO, USA) | \| 180.1 🡪163.1 \| \| --- \| \| 180.1 🡪105 \| | 14 | 6.5 | 13 | 24 |
| MDMA | CNS stimulant | Merck Life Science (St. Louis, MO, USA) | \| 194.1 🡪163 \| \| --- \| \| 194.1 🡪133.2 \| | 40.5 | 10 | 16.2 | 23 |
| Methadone | Opioid | Merck Life Science (St. Louis, MO, USA) | \| 310.2 🡪265 \| \| --- \| \| 310.2 🡪219.1 \| | 31 | 9 | 20.3 | 19 |
| Methandrostanolone | AAS | SelleckChem (Houston, TX, USA) | \| 301.1 🡪149.1 \| \| --- \| \| 301.1🡪283.2 \| | 70 | 7 | 21 | 17 |
| Methenolone | AAS | Toronto Research Chemicals (Toronto, Canada) | \| 303.0🡪187.2 \| \| --- \| \| 303.0🡪83 \| | 85 | 5 | 28 | 13 |
| Methyltestosterone | AAS | Merck Life Science (St. Louis, MO, USA) | \| 303.1🡪109.1 \| \| --- \| \| 303.1🡪97 \| | 160 | 7 | 33 | 13 |
| Morphine | Opioid | Merck Life Science (St. Louis, MO, USA) | \| 286.0🡪201.2 \| \| --- \| \| 286.0🡪165 \| | 101 | 4 | 35 | 17 |
| Nandrolone | AAS | NMI (North Ryde, NSW, Australia) | \| 275.1🡪257 \| \| --- \| \| 275.1🡪239.1 \| | 43 | 5 | 22 | 13 |
| Nicotine | Lifestyle drug and autonomic ganglia stimulant | Merck Life Science (St. Louis, MO, USA) | \| 163.1🡪117.2 \| \| --- \| \| 163.1🡪129.9 \| | 52 | 12 | 34 | 9 |
| Oxandrolone | AAS | NMI (North Ryde, NSW, Australia) | \| 307.1🡪289.1 \| \| --- \| \| 307.1🡪229.1 \| | 30 | 12 | 16 | 22 |
| Paracetamol | Analgesic agent | Merck Life Science (St. Louis, MO, USA) | \| 152.1🡪110.1 \| \| --- \| \| 152.1🡪93 \| | 74 | 10 | 23 | 12.8 |
| Paraxanthine | Metabolite of caffeine | Merck Life Science (St. Louis, MO, USA) | 181.1🡪123.8 | 86 | 11 | 27 | 19 |
| Ritalinic acid | CNS stimulant | Merck Life Science (St. Louis, MO, USA) | \| 220.1🡪84 \| \| --- \| \| 220.1🡪174.1 \| | 39 | 11.5 | 25 | 13 |
| Salbutamol | β-2 agonist | Merck Life Science (St. Louis, MO, USA) | \| 240.2🡪148.1 \| \| --- \| \| 240.2🡪166.2 \| | 35 | 5 | 25 | 12 |
| Sildenafil | PDE-5 inhibitor | Merck Life Science (St. Louis, MO, USA) | \| 475.0🡪283.1 \| \| --- \| \| 475.0🡪311 \| | 80 | 5 | 51 | 19 |
| Stanozolol | AAS | Merck Life Science (St. Louis, MO, USA) | \| 329.2🡪121.1 \| \| --- \| \| 329.2🡪203.1 \| | 50 | 10 | 49 | 19 |
| Tamoxifen | SERM | Merck Life Science (St. Louis, MO, USA) | \| 372.2🡪72 \| \| --- \| \| 372.2🡪129.2 \| | 228 | 7 | 51 | 10 |
| Testosterone | Endogenous steroid | Merck Life Science (St. Louis, MO, USA) | \| 289.4🡪97.1 \| \| --- \| \| 289.4🡪109.1 \| | 70 | 5 | 30 | 12 |
| Testosterone glucuronic acid | Phase II metabolite of testosterone | NMI (North Ryde, NSW, Australia) | \| 465.5🡪97.1 \| \| --- \| \| 465.5🡪109.1 \| | 100 | 5 | 38 | 11 |
| Testosterone sulphate | Phase II metabolite of testosterone | NMI (North Ryde, NSW, Australia) | \| 369.4🡪97.0 \| \| --- \| \| 369.4🡪109.1 \| | 135 | 6 | 32 | 12 |
| THC | Cannabis | Merck Life Science (St. Louis, MO, USA) | \| 315.1🡪193.3 \| \| --- \| \| 315.1🡪123.2 \| | 54 | 7 | 31 | 12 |
| THC-COOH | Metabolite of THC | Merck Life Science (St. Louis, MO, USA) | \| 345.1🡪326.7 \| \| --- \| \| 345.1🡪299.4 \| | 9 | 9 | 19 | 18.5 |
| Trenbolone | AAS | NMI (North Ryde, NSW, Australia) | \| 271.1🡪199 \| \| --- \| \| 271.1🡪237 \| | 60 | 10 | 32.3 | 16 |
| Cocaine-D3 | Deuterated standard for cocaine | Cerilliant (Austin, TX, USA) | \| 307.0🡪185.1 \| \| --- \| \| 307.0🡪153 \| | 135 | 10 | 48 | 13 |
| Creatinine-D3 | Deuterated standard for creatinine | Cerilliant (Austin, TX, USA) | \| 117.1🡪47.1 \| \| --- \| \| 117.1🡪73.1 \| | 135 | 10 | 48 | 13 |
| Epitestosterone-D3 | Deuterated standard for epitestosterone | NMI (North Ryde, NSW, Australia) | \| 292.0🡪97 \| \| --- \| \| 292.0🡪109 \| | 51 | 9 | 30 | 12 |
| MDMA-D5 | Deuterated standard for MDMA | Cerilliant (Austin, TX, USA) | \| 199.3🡪165.1 \| \| --- \| \| 199.3🡪135.1 \| | 50 | 6 | 17 | 20 |
| Morphine-D3 | Deuterated standard for morphine | Cerilliant (Austin, TX, USA) | \| 289.1🡪165 \| \| --- \| \| 289.1🡪153 \| | 100 | 6 | 51 | 19 |
| Nandrolone-D3 | Deuterated standard for nandrolone | NMI (North Ryde, NSW, Australia) | \| 278.0🡪109 \| \| --- \| \| 278.0🡪91 \| | 46 | 7 | 33 | 16 |
| Amphetamine-D5 | Deuterated standard for amphetamine | Cerilliant (Austin, TX, USA) | \| 141.2🡪124.1 \| \| --- \| \| 141.2🡪92.9 \| | 15 | 4.5 | 12 | 17 |
| Testosterone-D3 | Deuterated standard for testosterone | Merck Life Science (St. Louis, MO, USA) | \| 292.1🡪97.1 \| \| --- \| \| 292.1🡪109.1 \| | 103 | 6 | 30 | 11.5 |

Abbreviations used in this table:

AAS: Androgenic anabolic steroid

CNS: Central nervous system

EDDP: 2-ethylidene-1,5-dimethyl-3,3-diphenyl pyrrolidine

MDA: 3,4-Methylenedioxyamphetamine

MDMA: 3,4-Methyl​enedioxy​methamphetamine

NSAI: Nonsteroidal aromatase inhibitors

PDE-5: Phosphodiesterase type 5

SARM: Selective androgen receptor modulator

SERM: Selective estrogen receptor modulator

THC: Δ^9^-tetrahydrocannabinol

## Sample preparation

### Sample preparation for alcohol analysis

1 mL of a solution containing 2.5 g NaSO_3_, 30 µL 2-butanol and 10 µL tert-butanol in 2L MilliQ water was transferred to a head-space vial followed by the addition of 100 µL urine. The vial was capped and then ready for analysis. The method is used for routine alcohol analysis at the Department of Forensic Medicine, Section for Forensic Chemistry, Aarhus University [22].

### Sample preparation for drug analysis

400 µL urine was transferred to a 10 mL conical glass centrifuge tube followed by the addition of 40 µL β-glucuronidase, 200 µL 0.1 M ammonium acetate buffer (pH=6), and 40 µL internal standard solution 1 µg/mL in MeOH (amphetamine-d_5_, creatinine-d_3_, cocaine-d_3_, epitestosterone-d_3_, MDMA-d_5_, morphine-d_3_, nandrolone-d_3_, and testosterone-d_3_). The centrifuge tube was placed in a shaking incubator for 2 h at 48 ^o^C at 250 rpm. The sample was allowed to cool to room temperature, 300 µL 0.1 M HCl was added, and shaken for 30 seconds at 1400 rpm. 600 µL acetonitrile was then added and mixing for 30 seconds at 1650 rpm was performed. This step was subsequently repeated one more time. The sample was left for 15 min at ambient temperature for precipitation to occur. The sample was then centrifuged at 4000 rpm for 5 min. 2 mL of supernatant was transferred to a new 10 mL conical glass centrifuge tube. The supernatant was vaporized under a stream of 40 °C nitrogen and reconstituted using 400 µL MeOH in 0.1% formic acid (15:85, v/v). The sample was then shaken for 15 minutes at 1700 rpm. The reconstituted sample was finally divided into two vials, one for each LC-MS analysis.

## Analytical methods

### *Alcohol analysis using HS-GC-FID*

The ethanol concentration in urine was determined in a single determination by headspace gas chromatography using a well-established method as described in more detail elsewhere [22].

### *Data processing and analysis using UPLC-HR-qTOF-MS*

A previously described UPLC-HR-qTOF-MS-based method, which is used routinely for toxicological screening of biological samples, was used to analyze the urine samples [21]. The method was initially validated for a set of compounds in blood samples; nevertheless, it has demonstrated robustness when routinely applied for the analysis of urine samples as well. After the samples were analyzed, data processing and thereby also the peak identification was performed automatically by a post-run script and were subsequently reported in a CSV file format. Target substances were identified and reported from accurate-mass scan data using the software Target Analysis version 1.3 and Data Analysis version 4.1 from Bruker [21]. An in-house library consisting of 935 target substances containing the substance name, molecular formula, retention time, qualifier ion masses (if available), and individual minimum area. A part of this library consisting of all the reported compounds in this study is presented below in Table S1.2. Acceptance criteria for identification included a tolerance of +/- 5 mDa for [M+H]^+^, a tolerance of +/- 0.25 min for the retention time window of each target substance, the mass error (mass difference between measured and theoretical [M+H]^+^ values) < 5 ppm, the mSigma cut-off value was 250 (SigmaFitTM was used to calculate the mass errors for isotope peaks and relative abundances of the isotopes), and whether the presence of expected qualifier ions was included. Furthermore, a compound was included in the list, if the peak area was above 10,000 and if the peak intensity was above 1×10^5^. All detected compounds were verified using visual inspection of the EICs and fragmentation patterns if a fragmentation pattern was included for the compound of interest in the library as described below in Table S1.2.

**Table S1.2:** Compound information for the reported compounds using the LC-HR-qTOF-MS method

| m/z | rt | formula | name | CAS | minimum area | QI 1 | QI 2 | QI 3 | QI 1 min | QI 1 max | QI 2 min | QI 2 max | QI 3 min | QI 3 max | QI 1 SF | QI 2 SF | QI 3 SF |
| --- | --- | --- | --- | --- | --- | --- | --- | --- | --- | --- | --- | --- | --- | --- | --- | --- | --- |
| 315.231 | 8.86 | C21H30O2 | Tetrahydrocannabinol (THC) | CYQFCXCEBYINGO-IAGOWNOFSA-N | 2000.00 |  |  |  |  |  |  |  |  |  |  |  |  |
| 345.206 | 7.8 | C21H28O4 | Tetrahydrocannabinolsyre (THC-COOH) | YOVRGSHRZRJTLZ-HZPDHXFCSA-N | 2500.00 |  |  |  |  |  |  |  |  |  |  |  |  |
| 136.112 | 2.4 | C9H13N | Amphetamine | KWTSXDURSIMDCE-UHFFFAOYSA-N | 5000.00 | 91.054 |  |  | 0.001 | 1000 |  |  |  |  | C7H7 | C9H11 | - |
| 150.127 | 2.7 | C10H15N | Methamphetamine | MYWUZJCMWCOHBA-UHFFFAOYSA-N | 1500.00 | 91.054 |  |  | 0.001 | 1000 |  |  |  |  | C7H7 | C9H11 | - |
| 234.148 | 3.88 | C14H19NO2 | Methylphenidat | DUGOZIWVEXMGBE-UHFFFAOYSA-N | 3000.00 | 84.080 |  |  | 0.001 | 1000 |  |  |  |  | C5H10N | C12H16N | - |
| 220.133 | 3.32 | C13H17NO2 | Ritalinic acid | INGSNVSERUZOAK-UHFFFAOYSA-N | 5000.00 | 84.080 | 174.127 |  | 0.01 | 10 | 0.01 | 10 |  |  | C5H10N | C12H16N | C9H14N |
| 194.117 | 2.81 | C11H15NO2 | MDMA | SHXWCVYOXRDMCX-UHFFFAOYSA-N | 20000.00 | 135.044 |  |  | 0.01 | 4 |  |  |  |  | C8H7O2 | C10H11O2 | C10H11O2 |
| 180.101 | 2.62 | C10H13NO2 | MDA | NGBBVGZWCFBOGO-UHFFFAOYSA-N | 15000.00 | 135.044 |  |  | 0.3 | 4 |  |  |  |  | C8H7O2 | C10H11O2 | - |
| 304.154 | 4.25 | C17H21NO4 | Cocaine | ZPUCINDJVBIVPJ-LJISPDSOSA-N | 10000.00 | 182.117 |  |  | 0.001 | 1000 |  |  |  |  | C10H16NO2 | C9H12NO | C7H5O |
| 290.138 | 3.46 | C16H19NO4 | Benzoylecgonine | GVGYEFKIHJTNQZ-RFQIPJPRSA-N | 5000.00 | 168.101 |  |  | 0.001 | 1000 |  |  |  |  | C9H14NO2 | C7H5O | C9H12NO |
| 318.17 | 4.84 | C18H23NO4 | Cocaethylen | NMPOSNRHZIWLLL-XUWVNRHRSA-N | 5000.00 |  |  |  |  |  |  |  |  |  |  |  |  |
| 166.122 | 2.13 | C10H15NO | Ephedrin | KWGRBVOPPLSCSI-WPRPVWTQSA-N | 5000.00 |  |  |  |  |  |  |  |  |  |  |  |  |
| 370.164 | 4.21 | C21H23NO5 | Heroin | GVGLGOZIDCSQPN-PVHGPHFFSA-N | 5000.00 |  |  |  |  |  |  |  |  |  |  |  |  |
| 328.154 | 2.75 | C19H21NO4 | 6-MAM | JJGYGPZNTOPXGV-SSTWWWIQSA-N | 2000.00 | 211.075 |  |  | 0.001 | 1000 |  |  |  |  | C14H11O2 | C17H18NO | C14H9O |
| 286.143 | 1.6 | C17H19NO3 | Morphin | BQJCRHHNABKAKU-KBQPJGBKSA-N | 1500.00 | 229.085 |  |  | 0.001 | 1000 |  |  |  |  | C14H13O3 | C13H13O2 | - |
| 310.216 | 5.69 | C21H27NO | Methadon | USSIQXCVUWKGNF-UHFFFAOYSA-N | 1500.00 | 265.158 |  |  | 0.001 | 1000 |  |  |  |  | C19H21O | C16H15O | C7H5O |
| 278.190 | 5.41 | C20H23N | EDDP | AJRJPORIQGYFMT-PVOVUMCXSA-N | 2000.00 | 234.127 |  |  | 0.01 | 2 |  |  |  |  | C17H16N |  |  |
| 337.227 | 5.12 | C22H28N2O | Fentanyl | PJMPHNIQZUBGLI-UHFFFAOYSA-N | 1000.00 | 188.143 |  |  | 0.001 | 1000 |  |  |  |  | C13H18N | C8H9 | C14H18NO |
| 316.154 | 2.63 | C18H21NO4 | Oxycodon | BRUQQQPBMZOVGD-UHFFFAOYSA-N | 1500.00 | 298.143 |  |  | 0.001 | 1000 |  |  |  |  | C18H20NO3 | C15H15NO2 | C16H18NO2 |
| 264.195 | 3.91 | C16H25NO2 | Tramadol | TVYLLZQTGLZFBW-UHFFFAOYSA-N | 3000.00 |  |  |  |  |  |  |  |  |  | - | - | - |
| 250.180 | 4.01 | C15H23NO2 | Tramadol. N-demethyl- | VUMQHLSPUAFKKK-UHFFFAOYSA-N | 2000.00 |  |  |  |  |  |  |  |  |  |  |  |  |
| 250.180 | 2.8 | C15H23NO2 | Tramadol. O-demethyl- | UWJUQVWARXYRCG-UHFFFAOYSA-N | 3000.00 |  |  |  |  |  |  |  |  |  | C14H22NO2 | - | - |
| 300.159 | 2.34 | C18H21NO3 | Codein | OROGSEYTTFOCAN-DNJOTXNNSA-N | 4000.00 | 243.101 |  |  | 0.001 | 1000 |  |  |  |  | C15H15O3 | C14H15O2 | C15H13O2 |
| 285.078 | 6.43 | C16H13ClN2O | Diazepam | AAOVKJBEBIDNHE-UHFFFAOYSA-N | 1500.00 | 228.057 |  |  | 0.001 | 1000 |  |  |  |  | C14H11ClN | C15H14ClN2 | C14H11ClN |
| 316.048 | 5.92 | C15H10ClN3O3 | Clonazepam | DGBIGWXXNGSACT-UHFFFAOYSA-N | 350.00 |  |  |  |  |  |  |  |  |  |  |  |  |
| 389.112 | 3.8 | C17H17ClN6O3 | Zopiclon | GBBSUAFBMRNDJC-UHFFFAOYSA-N | 1500.00 | 245.022 |  |  | 0.001 | 1000 |  |  |  |  | C11H6ClN4O | C10H6ClN | C5H5ClNO |
| 308.175 | 4.57 | C19H21N3O | Zolpidem | ZAFYATHCZYHLPB-UHFFFAOYSA-N | 1500.00 | 236.130 |  |  | 0.04 | 3 |  |  |  |  | C16N2 | C17H15N2OS | - |
| 338.149 | 3.26 | C19H19N3O3 | Zolpidem. Phenyl-4-COOH | FELZONDEFBLTSP-UHFFFAOYSA-N | 2000.00 | 293.091 |  |  | 0.02 | 2 |  |  |  |  | C17H13N2O3 | |  |
| 296.103 | 6.1 | C16H13N3O3 | Nimetazepam | GWUSZQUVEVMBPI-UHFFFAOYSA-N | 2000.00 | 250.110 |  |  | 0.01 | 5 |  |  |  |  | C16H14N2O | |  |
| 343.051 | 6.03 | C17H12Cl2N4 | Triazolam | JOFWLTCLBGQGBO-UHFFFAOYSA-N | 1000.00 | 315.032 |  |  | 0.001 | 1000 |  |  |  |  | C16H11Cl2N3 | - | - |
| 359.046 | 5.75 | C17H12Cl2N4O | Triazolam. alpha-hydroxy- | BHUYWUDMVCLHND-UHFFFAOYSA-N | 2000.00 | 341.035 |  |  | 0.05 | 3 |  |  |  |  | C17H11Cl2N4 | |  |
| 300.089 | 4.75 | C16H14ClN3O | Chlordiazepoxid | ANTSCNMPPGJYLG-UHFFFAOYSA-N | 1000.00 |  |  |  |  |  |  |  |  |  |  |  |  |
| 287.058 | 5.84 | C15H11ClN2O2 | Oxazepam | ADIMAYPTOBDMTL-UHFFFAOYSA-N | 1500.00 | 231.068 |  |  | 0.03 | 5 |  |  |  |  | C13H12ClN2 | C14H10ClN2 | C15H10ClN2O |
| 309.090 | 5.96 | C17H13ClN4 | Alprazolam | VREFGVBLTWBCJP-UHFFFAOYSA-N | 2000.00 |  |  |  |  |  |  |  |  |  | C16H12ClN3 | C15H12ClN2 | C10H9ClN3 |
| 325.085 | 5.74 | C17H13ClN4O | Alprazolam. alpha-hydroxy- | ZURUZYHEEMDQBU-UHFFFAOYSA-N | 2000.00 | 279.068 | 307.074 |  | 0.01 | 2 | 0.01 | 2 |  |  | C17H12ClN2 | C17H12ClN4 | |
| 325.171 | 5.28 | C20H21FN2O | Citalopram | WSEQXVZVJXJVFP-UHFFFAOYSA-N | 3000.00 | 262.102 |  |  | 0.001 | 1000 |  |  |  |  | C18H13FN | C7H6F | C17H10FN |
| 325.171 | 5.28 | C20H21FN2O | Escitalopram | WSEQXVZVJXJVFP-FQEVSTJZSA-N | 1200.00 | 262.102 |  |  | 0.001 | 1000 |  |  |  |  | C18H13FN | C7H6F | C17H10FN |
| 384.174 | 5.07 | C21H25N3O2S | Quetiapin | URKOMYMAXPYINW-UHFFFAOYSA-N | 2000.00 | 253.079 |  |  | 0.001 | 1000 |  |  |  |  | C15H13N2S | C17H15N2S | C13H8NS |
| 316.092 | 5.84 | C18H18ClNS | Chlorprothixen | WSPOMRSOLSGNFJ-AUWJEWJLSA-N | 600.00 | 231.003 |  |  | 0.001 | 1000 |  |  |  |  | C13H8ClS | C16H12ClS | C14H10ClS |
| 370.179 | 3.26 | C17H27N3O4S | Amisulprid | NTJOBXMMWNYJFB-UHFFFAOYSA-N | 2000.00 | 242.048 | 112.112 |  | 0.1 | 3 | 0.01 | 2 |  |  |  |  |  |
| 313.148 | 2.51 | C17H20N4S | Olanzapin | KVWDHTXUZHCGIO-UHFFFAOYSA-N | 2500.00 | 256.090 |  |  | 0.001 | 1000 |  |  |  |  | C14H14N3S | C12H9N2S | C16H16N3S |
| 448.155 | 5.61 | C23H27Cl2N3O2 | Aripiprazol | CEUORZQYGODEFX-UHFFFAOYSA-N | 500.00 | 285.092 |  |  | 0.001 | 1000 |  |  |  |  | C14H19Cl2N2 | C13H16NO2 | C10H10NO2 |
| 310.141 | 5.8 | C17H18F3NO | Fluoxetin | RTHCYVBBDHJXIQ-UHFFFAOYSA-N | 1500.00 |  |  |  |  |  |  |  |  |  | - | - | - |
| 306.081 | 5.8 | C17H17Cl2N | Sertralin | VGKDLMBJGBXTGI-UHFFFAOYSA-N | 1500.00 | 158.976 |  |  | 0.001 | 1000 |  |  |  |  | C7H5Cl2 | C10H9 | C16H13Cl2 |
| 330.15 | 5.49 | C19H20FNO3 | Paroxetin | AHOUBRCZNHFOSL-UHFFFAOYSA-N | 1500.00 | 192.118 |  |  | 0.001 | 1000 |  |  |  |  | C12H15FN | C11H12F | C8H7O3 |
| 275.200 | 6.1 | C18H26O2 | Nandrolone | NPAGDVCDWIYMMC-IZPLOLCNSA-N | 9000000.00 | |  |  |  |  |  |  |  |  |  |  |  |
| 277.216 | 6.87 | C18H28O2 | 19-Norandrosteron | UOUIARGWRPHDBX-CQZDKXCPSA-N | 5000.00 | 259.206 | 95.085 |  | 0.01 | 10 | 0.01 | 10 |  |  |  | C18H27O | C7H11 |
| 277.216 | 6.79 | C18H28O2 | 19-Noretiocholanolon | UOUIARGWRPHDBX-DHMVHTBWSA-N | 5000.00 | 259.206 |  |  | 0.01 | 10 |  |  |  |  | C18H27O |  |  |
| 287.200 | 5.88 | C19H26O2 | Boldenone | RSIHSRDYCUFFLA-DYKIIFRCSA-N | 2000.00 |  |  |  |  |  |  |  |  |  |  |  |  |
| 289.216 | 6.65 | C19H28O2 | 5beta-androst-1-en-17beta-ol-3-one | OKJCFMUGMSVJBG-WYSACVJLSA-N | 4000.00 | 289.217 | 271.205 | 121.064 |  |  |  |  |  |  | C19H29O2(+) | C19H27O(+) | C8H9O(+) |
| 271.169 | 5.95 | C18H22O2 | Trenbolone | MEHHPFQKXOUFFV-OWSLCNJRSA-N | 4000.00 |  |  |  |  |  |  |  |  |  |  |  |  |
| 271.169 | 6.08 | C18H22O2 | 17-alpha-trenbolone | MEHHPFQKXOUFFV-XDNAFOTISA-N | 2000.00 | 253.158 |  |  | 0.01 | 10 |  |  |  |  | C18H21O |  |  |
| 303.232 | 6.57 | C20H30O2 | Methenolone | ANJQEDFWRSLVBR-VHUDCFPWSA-N | 4000.00 | 303.232 | 285.221 | 97.064 | 0.01 | 10 | 0.01 | 10 | 0.01 | 10 | C20H31O2(+) | C20H29O(+) | C6H9O(+) |
| 475.212 | 5.27 | C22H30N6O4S | Sildenafil | BNRNXUUZRGQAQC-UHFFFAOYSA-N | 1000.00 |  |  |  |  |  |  |  |  |  | - | - | - |
| 294.171 | 5.76 | C17H19N5 | Anastrozole | YBBLVLTVTVSKRW-UHFFFAOYSA-N | 2000.00 |  |  |  |  |  |  |  |  |  |  |  |  |
| 240.159 | 1.83 | C13H21NO3 | Salbutamol | NDAUXUAQIAJITI-UHFFFAOYSA-N | 1500.00 |  |  |  |  |  |  |  |  |  |  |  |  |
| 163.123 | 0.88 | C10H14N2 | Nicotine | SNICXCGAKADSCV-JTQLQIEISA-N | 5000.00 |  |  |  |  |  |  |  |  |  |  |  |  |
| 177.102 | 1.21 | C10H12N2O | Cotinine | UIKROCXWUNQSPJ-UHFFFAOYSA-N | 10000.00 | 98.06 |  |  | 0.05 | 5 |  |  |  |  | C5H8NO |  |  |
| 152.070 | 1.89 | C8H9NO2 | Paracetamol | RZVAJINKPMORJF-UHFFFAOYSA-N | 100000.00 | |  |  |  |  |  |  |  |  |  |  |  |
| 207.138 | 7.08 | C13H18O2 | Ibuprophen | HEFNNWSXXWATRW-UHFFFAOYSA-N | 20000.00 |  |  |  |  |  |  |  |  |  |  |  |  |
| 160.133 | 2.29 | C8H17NO2 | Pregabalin | AYXYPKUFHZROOJ-UHFFFAOYSA-N | 5000.00 |  |  |  |  |  |  |  |  |  |  |  |  |
| 238.099 | 3.41 | C13H16ClNO | Ketamine | YQEZLKZALYSWHR-UHFFFAOYSA-N | 1500.00 | 125.015 |  |  | 0.001 | 1000 |  |  |  |  | C7H6Cl | C11H12Cl | C13H15ClN |

### *Drug analysis using HPLC-MS/MS method*

The urine samples were analyzed for 62 compounds using a developed HPLC-MS/MS method. For details on the validation of the method, please see Supplementary Document 2.

#### Quality control samples

Three QC samples were used for each run to monitor the performance of the method.

*QC- instrument*: A 50 ng/mL solution containing all 62 substances listed above in Table S1.1 prepared in MeOH: Milli-Q water (15:85) with 0.1% formic acid was used to verify whether the retention time was within the detection window. Furthermore, the same sample was used to monitor the performance of the instrument. The QC-instrument solution was analyzed in every sample batch.

*QC-MilliQ-water*: 400 µL Milli-Q water was analyzed using the sample preparation method described in section 2.3.2. The QC-Milli-Q water sample was used to monitor potential contamination in the lab or from the solvents used in the sample preparation.

*QC-urine*: 400 µL QC-urine sample was analyzed using the sample preparation method described in section 2.3.2. The QC-urine sample was used for verification of the extraction procedure and to monitor instrument performance.

#### Instrumental settings HPLC-MS/MS

Targeted analysis of the 62 compounds was performed on a Sciex ExionLC^TM^ with a Sciex QTRAP® 6500+ (QTRAP). All compounds were analyzed in ESI^+^ mode. A direct infusion was used to select and optimize two multiple reaction monitoring (MRM) transitions per compound. This included optimization of the declustering potential (DP), entrance potential (EP), collision energy (CE), and collision exit cell potential (CXP). The optimized parameters for each compound transition can be found in Table S1.1. The analytical column was a Phenomenex Kinetex® C18 with a particle size of 1.7 µm and dimensions of 2.1 x 100 mm (Phenomenex, Lane Cove West, NSW, Australia). In addition, an ACQUITY Column In-Line filter kit (Waters™) containing a holder and 0.2 µm stainless steel filters was installed to protect the column and instrument components from a build-up of particulate matter. The samples were stored in an autosampler with a temperature of 5 °C. The column temperature was 45 °C, the flow rate of the method was 0.3 mL/min, and the injection volume was 10 µL. The QTRAP was operating in positive electrospray ionization mode (ESI^+^) at a temperature of 550 ºC with an ion spray voltage of 4500 V. The mobile phases were 0.1 % formic acid in milliQ water for mobile phase A and 50:50 MeOH/acetonitrile with 0.1 % formic acid for mobile phase B. The gradient program was as follows: 2.5 % B hold for 3.5 minutes, 2.5-5% B (3.5-8 min, linearly), 5-40% B (8-16 min, linearly), 40% B hold for 6 minutes, 40-50% B (22-30 min, linearly), 50-100% B (30-31 min, linearly) followed by equilibration to initial gradient settings for 9 minutes for a total analytical run of 40 minutes. Acquired data was processed utilizing MultiQuant 3.0.2 software (SCIEX) and the integration of peaks was subsequently transferred to a CSV file. A substance was considered detected if its retention time matched the expected value based on the QC-instrument sample, both MRM transitions were detected, and the ratio between the qualifier ions was as expected.

### Data analysis

Microsoft Excel version 16.0.5266.1000 was used for data storage of the CSV files. Data analysis was made in RStudio version 1.2.1335 with R version 4.1.2. A Chi-square test of independence was performed to examine whether there was a significant difference between the use of drugs and alcohol between victims and perpetrators and to see if there was a significant difference between the different types of perpetrators. All chi-square test calculations were performed on data normalized to the number of people for each category. When p-values were assessed between the different categories Bonferroni correction was used.
