# Supplementary material for: Detection of substance use in clinical forensic cases: urine analysis of victims and perpetrators
Source: Forensic Sci Med Pathol. 2024 Sep 5;21(2):522–31. doi: 10.1007/s12024-024-00873-w (PMC12325547; doi:10.1007/s12024-024-00873-w)
Supplement: Supplementary file 3 — Supplementary Material 3 [file 12024_2024_873_MOESM3_ESM.docx]

Supplementary document 2

Method validation of the LC-MS/MS method

Validation of the method was performed qualitatively for 62 of the targeted compounds and quantitatively for eight of the compounds, namely amphetamine, cocaine, creatinine, heroin, MDMA, morphine, testosterone, epitestosterone, and nandrolone. The evaluated parameters included linearity, selectivity, limit of detection (LOD), matrix effects, recovery, and robustness. Additional parameters were evaluated for the eight compounds with a deuterated internal standard, namely the lower limit of quantitation (LLOQ), the accuracy, and the precision. All calculations performed for compounds with a deuterated internal standard were based on the ratio between the peak areas of the native standard to the area of the deuterated internal standards. The calculations for the remaining compounds were performed based on the peak area. Tables summarizing the results presented in each subsection are shown at the end of the document.

#### Selectivity

The selectivity of the 62 selected compounds was examined using 10 blank urine samples and 10 urine samples spiked by all compounds to a concentration of 200 ng/mL. The samples included five male and five female urine samples collected on different days and times of the day ensuring a variable urine concentration. An examination of overlapping compounds was made by a visual comparison of the extracted ion chromatograms and if doubt, the resolution was calculated (see equation 1). In equation 1, $\Delta t_{r}$ is the separation between peaks in units of time and $w_{\frac{1}{2}av}$ is the width at half-height of the peaks [1]. An acceptable separation between the two peaks of interest responded to a resolution value above 2.

$$Resolution=\frac{0.589\Delta t_{r}}{w_{\frac{1}{2}av}} (1\boldsymbol{)}$$

It was possible to separate most of the compounds with a resolution >2. Those that had an overlap were however possible to separate based on the precursor ions. When comparing the blank urine with the spiked samples, 10 compounds (testosterone, epitestosterone, caffeine, creatinine, DHEA, epitestosterone sulfate, paracetamol, paraxanthine, and testosterone sulfate) were present in the blank urine samples, and a determination of the selectivity in the matrix was therefore not possible.

#### Linearity, LOD, LLOQ, and carryover

The linearity was assessed through a calibration curve with intercept (0,0) and a weighting of 1/x made up of eight concentration levels (0, 10, 25, 50, 100, 150, 250, and 500 ng/mL) with a fixed level at 40 ng/mL deuterated internal standards added to each concentration level. The R^2^ values were calculated using a *goodness of fit*-analysis.

The acceptance criterium for the linearity assessment was R^2^>0.99. LOD and LOQ were estimated based on 10 spiked urine samples at three concentration levels (2 ng/mL, 10 ng/mL, and 50 ng/mL). LOD and LLOQ were calculated as 3 and 10 times the standard deviation of the response between samples at the lowest concentration level, where peaks from both MRM transitions were still visually distinct. Carryover was tested by analyzing three blank samples after a high-level sample containing 500 ng/mL and the acceptance criterium was below 0.1% of the compound being present in the blank when compared to the sample at 500 ng/mL.

The majority of compounds included in the method showed linearity with an R^2^-value≥0.99. Only four (2α-methyl-5α-androstan-3α-ol-17-one, clomiphene, tamoxifen, and THC-COOH) had a lower R^2^-value and were in the range from 0.95 to 0.98. The LOD was calculated for all and was in the range of 0.03 to 141ng/mL. However, as ten compounds were already present in the unspiked urine, LOD was then estimated based on the lowest visual concentration in the linearity study, and the matrix effects were included. LLOQ values for the eight compounds were between 0.9 to 16.1 ng/mL. Furthermore, the majority of compounds included in the method were below 0.1 in the first blank after the 500 ng/mL high concentration. However, for 16β-hydroxystanozolol which had a carryover in the first blank above 0.1, no carryover was detected in the 2nd and 3rd blanks. For creatinine, the level of carryover remained the same in all three following blank samples, and for tamoxifen, it was at 0.346 in the 2nd blank and 0.116 in the 3rd. To see all the results, please see Supplementary document Table 1 below.

**Supplementary document Table 1:** Validation results for all compounds included in the method.

| Compound | rt (min) ± %RSD  (N=9) | Linearity, R^2^ | Lineary range (ng/mL) | Carryover  (1st blank) | LOD  (N=10)  (ng/mL) | Recovery % ± SD  (N=10) | Matrix effect (%)  (N=10) |
| --- | --- | --- | --- | --- | --- | --- | --- |
| 16β-hydroxystanozolol | 21.11 ± 0.06 | 0.997 | 0-1000 | 0.12* | 7.1 | - | - |
| 17-epioxandrolone | 29.51 ± 0.03 | 0.998 | 0-1000 | <0.1 | 1.5 | \| 74.66 ± 11.95 \| \| --- \| | 20.05 ± 7.34 |
| 17-α-trenbolone | 22.07 ± 0.03 | 0.994 | 0-250 | <0.1 | 22.4 | \| 84.59 ± 5.67 \| \| --- \| | 22.54 ± 10.89 |
| 17α-methyl-5α-androstane-3α,17β-diol | 31.83 ± 0.02 | 0.989 | 0-100 | <0.1 | 6.4 | 122.02 ± 55.03 | 51.57 ± 14.43 |
| 17α-methyl-5β-androstane-3α,17β-diol | 31.36 ± 0.04 | 0.99 | 0-1000 | <0.1 | 3.1 | 71.82 ± 9.88 | 12.25 ± 8.94 |
| 19-norandrosterone | 31.09 ± 0.04 | 0.999 | 0-1000 | <0.1 | 1.1 | \| 79.68 ± 6.22 \| \| --- \| | 18.15 ± 3.44 |
| 19-noretiocholanolone | 29.96 ± 0.04 | 0.999 | 0-1000 | <0.1 | 1.8 | \| 79.07 ± 7.77 \| \| --- \| | 21.57 ± 3.72 |
| 2α-methyl-5α-androstan-3α-ol-17-one | 32.19 ± 0.02 | 0.979 | 0-1000 | <0.1 | 141.3 | \| 56.97 ± 24.49 \| \| --- \| | 24.80 ± 17.82 |
| 1-Methylene-5α-androstan-3α-ol-17-one | 31.77 ± 0.02 | 0.988 | 0-500 | <0.1 | 51.2 | \| 100.77 ± 38.48 \| \| --- \| | 58.99 ± 7.76 |
| 3'-hydroxystanozolol | 21.94 ± 0.05 | 0.998 | 0-1000 | <0.1 | 0.5 | 81.81 ± 6.98 | 23.17 ± 4.09 |
| 4-hydroxytamoxifen | 26.53 ± 0.04 | 0.998 | 0-1000 | <0.1 | 0.7 | 77.56 ± 8.78 | 12.73 ± 6.64 |
| 5β-androst-1-en-17β-ol-3-one | 28.47 ± 0.04 | 0.998 | 0-500 | <0.1 | 6.7 | 75.85 ± 13.25 | 26.87 ± 9.10 |
| 5β-androst-1-en-3α-ol-17-one | 31.37 ± 0.04 | 0.987 | 0-1000 | <0.1 | 30.8 | 78.13 ± 16.28 | 18.58 ± 12.33 |
| 9α-fluoro-17α-methyl-4-androsten-3α, 6β, 11β,17β-tetrol | 13.59 ± 0.02 | 0.998 | 0-500 | <0.1 | 5.7 | 80.53 ± 12.28 | 84.97 ± 1.75 |
| Amphetamine | 7.56 ± 0.09 | 0.998 | 0-1000 | <0.1 | 4.8 | 97.00 ± 13.54 | 51.53 ± 12.79 |
| Anastrozole | 18.01 ± 0.02 | 0.986 | 0-100 | <0.1 | 0.6 | 81.45 ± 8.86 | 49.89 ± 4.04 |
| Benzoylecgonine | 13.25 ± 0.02 | 0.998 | 0-100 | <0.1 | 0.2 | 82.12 ± 9.44 | 25.71 ± 4.58 |
| Boldenone | 22.46 ± 0.04 | 0.992 | 0-250 | <0.1 | 1.8 | 78.44 ± 4.36 | 31.79 ± 4.18 |
| Caffeine | 12.06 ± 0.03 | 0.999 | 0-100 | <0.1 | 0.6 | 76.18 ± 9.50 | 55.23 ± 5.47 |
| Citalopram | 17.11 ± 0.01 | 0.998 | 0-100 | <0.1 | 0.03 | 78.40 ± 13.77 | 54.31 ± 4.30 |
| Clenbuterol | 13.85 ± 0.01 | 0.998 | 0-100 | <0.1 | 0.3 | 80.16 ± 13.05 | 45.12 ± 5.04 |
| Clomiphene | 31.72 ± 0.02 | 0.976 | 0-500 | <0.1 | 1.9 | 81.36 ± 21.42 | 47.30 ± 8.84 |
| Cocaethylene | 15.66 ± 0.01 | 0.99 | 0-50 | <0.1 | 0.4 | 77.46 ± 13.11 | 49.39 ± 4.23 |
| Cocaine | 14.46 ± 0.02 | 0.996 | 0-100 | <0.1 | 1.1 | 76.18 ± 8.43 | 39.71 ± 4.29 |
| Cotinine | 1.65 ± 0.02 | 0.996 | 0-500 | <0.1 | 1.0 | 74.74 ± 7.47 | 68.31 ± 2.75 |
| Creatinine | 0.85 ± 0 | 0.986 | 0-1000 | 3.47* | 0.32 | 99.35 ± 4.47 | -18.81 ± 358.54 |
| DHEA | 27.72 ± 0.03 | 0.998 | 0-500 | <0.1 | 0.5 | 79.08 ± 10.50 | 17.09 ± 6.44 |
| Diazepam | 24.03 ± 0.06 | 0.999 | 0-250 | <0.1 | 0.2 | 79.09 ± 6.53 | 13.29 ± 6.28 |
| EDDP | 17.50 ± 0.01 | 0.999 | 0-100 | <0.1 | 0.2 | 81.24 ± 9.91 | 30.07 ± 6.38 |
| Enobosarm | 28.97 ± 0.04 | 0.998 | 0-250 | <0.1 | 0.3 | 76.73 ± 8.24 | 20.70 ± 5.23 |
| Ephedrine | 5.47 ± 0.08 | 0.993 | 0-500 | <0.1 | 18.2 | 76.64 ± 7.36 | 36.78 ± 9.69 |
| Epitestosterone | 28.95 ± 0.04 | 0.997 | 0-250 | <0.1 | 0.42 | 84.63 ± 10.63 | 3.77 ± 7.40 |
| Epitestosterone glucuronic acid | 24.18 ± 0.05 | 0.999 | 0-1000 | <0.1 | - | - | 9.74 ± 5.41 |
| Epitestosterone sulfate | 20.44 ± 0.04 | 0.999 | 0-1000 | <0.1 | 0.5 | 81.64 ± 8.48 | 31.76 ± 5.41 |
| Flunitrazepam | 19.97 ± 0.02 | 0.993 | 0-250 | <0.1 | 0.5 | 69.78 ± 8.48 | 33.77 ± 5.54 |
| Fluoxymesterone | 21.12 ± 0.03 | 0.994 | 0-100 | <0.1 | 0.3 | 81.06 ± 7.58 | 20.41 ± 6.49 |
| Heroin | 14.38 ± 0.02 | 0.994 | 0-500 | <0.1 | 0.8 | 70.50 ± 10.91 | 49.97 ± 4.22 |
| Letrozol | 17.85 ± 0.01 | 0.999 | 0-250 | <0.1 | 0.6 | 80.43 ± 10.69 | 53.16 ± 3.41 |
| Ligandrol | 31.60 ± 0.02 | 0.995 | 0-1000 | <0.1 | 0.9 | 87.24 ± 17.50 | -5.27 ± 17.74 |
| MDA | 10.09 ± 0.09 | 0.999 | 0-1000 | <0.1 | 1.2 | 96.05 ± 12.15 | 50.87 ± 14.27 |
| MDMA | 11.19 ± 0.05 | 0.999 | 0-250 | <0.1 | 0.3 | 81.15 ± 9.39 | 41.05 ± 2.51 |
| Methadone | 19.70 ± 0.02 | 0.997 | 0-100 | <0.1 | 0.3 | 80.65 ± 5.34 | 30.52 ± 3.57 |
| Methandrostanolone | 24.83 ± 0.04 | 0.997 | 0-250 | <0.1 | 0.2 | 78.47 ± 10.71 | 23.26 ± 5.62 |
| Methenolone | 29.06 ± 0.04 | 0.992 | 0-500 | <0.1 | 0.2 | 75.00 ± 16.23 | 15.13 ± 10.45 |
| Methyltestosterone | 28.49 ± 0.04 | 0.997 | 0-250 | <0.1 | 0.3 | 76.71 ± 11.80 | 21.62 ± 7.07 |
| Morphine | 2.77 ± 0.04 | 0.996 | 0-500 | <0.1 | 2 | 77.36 ± 13.91 | 58.69 ± 4.87 |
| Nandrolone | 23.25 ± 0.04 | 0.999 | 0-250 | <0.1 | 1.8 | 78.53 ± 6.88 | 33.02 ± 3.75 |
| Nicotine | 1.48 ± 0.04 | 0.995 | 0-250 | <0.1 | 11.2 | 73.12 ± 9.53 | 46.56 ± 4.49 |
| Oxandrolone | 24.10 ± 0.04 | 0.995 | 0-500 | <0.1 | 1.1 | 77.94 ± 9.72 | 24.23 ± 5.96 |
| Paracetamol | 4.44 ± 0.06 | 0.997 | 0-1000 | <0.1 | 3.5 | 71.66 ± 14.41 | 72.81 ± 3.75 |
| Paraxanthine | 8.77 ± 0.12 | 0.999 | 0-1000 | <0.1 | 0.7 | 64.43 ± 16.30 | 63.46 ± 23.81 |
| Ritalinic acid | 12.79 ± 0.02 | 0.996 | 0-100 | <0.1 | 10.9 | 78.44 ± 8.83 | 28.27 ± 4.81 |
| Salbutamol | 6.05 ± 0.13 | 0.998 | 0-250 | <0.1 | 0.8 | 78.73 ± 7.54 | 22.69 ± 14.86 |
| Sildenafil | 17.97 ± 0.01 | 0.996 | 0-500 | <0.1 | 0.4 | 78.93 ± 12.34 | 43.12 ± 7.76 |
| Stanozolol | 29.24 ± 0.08 | 0.996 | 0-1000 | <0.1 | 1.6 | 77.57 ± 7.46 | 20.86 ± 6.67 |
| Tamoxifen | 31.85 ± 0.01 | 0.982 | 0-1000 | 0.548 | 1.7 | 67.63 ± 24.73 | 52.65 ± 11.73 |
| Testosterone | 25.89 ± 0.04 | 0.997 | 0-250 | <0.1 | 0.46 | 104.73 ± 10.35 | 13.76 ± 6.03 |
| Testosterone glucuronic acid | 19.77 ± 0.02 | 0.999 | 0-1000 | <0.1 | - | - | 25.20 ± 4.90 |
| Testosterone sulfate | 19.51 ± 0.03 | 0.999 | 0-1000 | <0.1 | 0.5 | 93.66 ± 6.38 | 33.05 ± 5.42 |
| THC | 32.49 ± 0.01 | 0.993 | 0-1000 | 0.208* | 1.4 | 76.15 ± 16.71 | 20.77 ± 5.54 |
| THC-COOH | 32.23 ± 0.02 | 0.984 | 0-1000 | <0.1 | 15.6 | 78.97 ± 18.60 | 64.35 ± 15.05 |
| Trenbolone | 21.30 ± 0.03 | 0.999 | 0-100 | <0.1 | 1.3 | 76.91 ± 6.78 | 39.75 ± 4.13 |
| Cocaine-D3 | 14.45 ± 0.02 | 0.997 | 0-250 | <0.1 | - | - | - |
| Creatinine-D3 | 0.81 ± 0.01 | 0.999 | 0-1000 | <0.1 | - | - | - |
| Epitestosterone-D3 | 28.80 ± 0.04 | 0.999 | 0-1000 | <0.1 | - | - | - |
| MDMA-D5 | 11.12 ± 0.06 | 0.999 | 0-1000 | <0.1 | - | - | - |
| Morphine-D3 | 2.73 ± 0.04 | 0.998 | 0-1000 | <0.1 | - | - | - |
| Nandrolone-D3 | 23.12 ± 0.04 | 0.993 | 0-250 | <0.1 | - | - | - |
| Amphetamine-D5 | 7.40 ± 0.09 | 0.997 | 0-250 | <0.1 | - | - | - |
| Testosterone-D3 | 25.78 ± 0.04 | 0.994 | 0-100 | <0.1 | - | - | - |

*For 16β-hydroxystanozolol no carryover was detected in the 2^nd^ and 3^rd^ blank. For creatinine. the level of carryover remained the same in all blanks and for tamoxifen, it was at 0.346 in the 2^nd^ blank and 0.116 in the 3^rd^.

#### Recovery and Matrix effects

The recovery and matrix effects were determined based on 10 different urine samples spiked in a 200 ng/mL level for all 62 compounds, and the same 10 samples without any spiked compounds. The urine samples were obtained from both men and women and were collected at different times during the day to ensure variation in the matrix. The matrix effect and the recovery was calculated using equation 2 and 3.

$$Matrix effects = \frac{(D-C+A) \times100}{D} (2)$$

$$Recovery = \frac{(B-A) \times100}{(C-A)} (3)$$

In equations 2 and 3, A is the peak area of a compound in the unspiked sample, B is the peak area of a compound in spiked samples C is the peak area of a compound in a sample spiked after protein precipitation, and D is the peak area of a compound in pure standard solution containing the compounds in the spike level. For each specific compound, please see Supplementary document Table 2. As seen in Supplementary document Table 2, the standard deviation of creatinine is close to 20 times higher than the matrix effect value. This is a result of creatinine being both concentrated and by experience we also saw that it stuck to the column material.

**Supplementary document Table 2:** Validation results for the quantitative compounds included in the method.

| Compound | LLOQ  (N=10)  (ng/mL) | Precision (N=3) | | | | | | % Accuracy ±SD | | |
| --- | --- | --- | --- | --- | --- | --- | --- | --- | --- | --- |
|  |  | RSD_r_ (%) | | | RSD_R_ (%) | | |  |  |  |
|  |  | (N=6)  50 ng/mL | (N=6)  200 ng/mL | (N=6)  400 ng/mL | (N=6)  50 ng/mL | (N=6)  200 ng/mL | (N=6)  400 ng/mL | (N=6)  50 ng/mL | (N=6)  200 ng/mL | (N=6)  400 ng/mL |
| Amphetamine | 16.1 | 8.4 | 3.1 | 2.7 | 7.6 | 6.9 | 6.6 | 101.6 ± 7.0 | 88.8 ± 5.1 | 88.5 ± 4.3 |
| Cocaine | 3.8 | 2.7 | 4.5 | 2.5 | 14.1 | 10.2 | 10.7 | 118.5 ± 14.5 | 106.6 ± 7.0 | 91.7 ± 6.5 |
| Creatinine | 10.0 | 7.2 | 10.9 | 7.0 | 12.7 | 15.3 | 8.4 | 124567.3 ± 117466.7 | 9769.0 ± 35737.3 | 2837.5 ± 15383.9 |
| Epitestosterone | 12.5 | 4.6 | 2.8 | 4.0 | 14.2 | 12.8 | 14.6 | 96.4 ± 9.2 | 98.5 ± 8.9 | 96.4 ± 9.2 |
| MDMA | 0.9 | 3.7 | 2.7 | 6.4 | 9.6 | 10.6 | 8.5 | 104.7 ± 8.6 | 102.1 ± 10.9 | 104.7 ± 8.6 |
| Morphine | 6.8 | 6.1 | 5.9 | 3.8 | 6.6 | 8.4 | 8.6 | 98.8 ± 8.3 | 102.3 ± 6.5 | 98.8 ± 8.3 |
| Nandrolone | 5.9 | 2.3 | 3.6 | 2.8 | 10.2 | 10.8 | 12.0 | 101.3 ± 8.2 | 100.5 ± 6.6 | 101.3 ± 8.2 |
| Testosterone | 6.25 | 4.1 | 1.9 | 2.4 | 5.5 | 6.6 | 10.3 | 91.2 ± 7.8 | 95.4 ± 9.0 | 91.2 ± 7.8 |

#### Accuracy and Precision

An examination of the accuracy and precision was made for the eight compounds used for a quantitative determination (amphetamine, cocaine, creatinine, epitestosterone, MDMA, morphine, nandrolone, and testosterone). The accuracy and precision were evaluated by spiking samples at three different concentration levels, namely 50 ng/mL (low), 200 ng/mL (medium), and 400 ng/mL (high). Analysis was performed in a series of six at the three concentration levels in duplicates. The accuracy was calculated as the percent recovery of compound in the spiked matrix and the precision was calculated as the relative standard deviation of the peak areas. The acceptance criteria for accuracy are 100% ± 15 % except at LOQ where it should be within 100% ± 20 %. The precision should be <15% except at LOQ where it should be <20%.

Accuracy and precision within the same day (intra-day, measured as RSD_r_) and precision between days (inter-day, measured as RSD_R_) were calculated for the eight quantitative compounds included in the method. Looking at the accuracy of the compounds in Supplementary document Table 2, seven out of the eight compounds fulfill the acceptance criteria. However, creatinine does not, and it is therefore not possible to include it in the method as a quantitative compound. Furthermore, the acceptance criteria for both intra-day and inter-day precision of the eight compounds were met by all target compounds.

#### Robustness

Stability was assessed for samples stored at room temperature reflecting the time from when samples were collected to the time point when they are placed in the freezer. Samples were prepared according to the sample preparation procedure described in section 2.3.2 of the article and analyzed to time points 0, 1, 3, and 24 hours and five days. The stability was also assessed on samples stored at -20 ^o^C and samples were prepared and analyzed to the time points 0, 1, 1.5, 2, and 3 months. Furthermore, the stability was tested for samples not being reconstituted after vaporization on the day of the sample preparation but stored in the freezer. Time points for this study included 0, 14, 28, 42 days and 2 months. Samples were then reconstituted in 400 µL methanol/0.1% formic acid (15:85, v/v) on the day of analysis. All urine samples used in this study included a pool of the ten urine samples used in the other parts of the method validation. The stability of the compounds was determined by calculating the percentage difference in the peak area ratio between the compounds and their respective deuterated standards at different time points, relative to the zero-time sample. Results are presented below in Supplementary document Table 3.

**Supplementary document Table 3:** Results from the stability study of the eight compounds in the method with an associated internal standard

|  | Time | Amphetamine | Cocaine | Creatinine | Epitestosterone | MDMA | Morphine | Nandrolone | Testosterone |
| --- | --- | --- | --- | --- | --- | --- | --- | --- | --- |
|  | 0 | 100+/-1.9 | 100+/-2.1 | 100+/-8.6 | 100+/-3.7 | 100+/-2.7 | 100+/-1.6 | 100+/-2.4 | 100+/-0.5 |
| Room temperature | 1 h | 100.9+/-4.7 | 100.3+/-4.7 | 87.7+/-2.8 | 108+/-5.1 | 101.8+/-4.5 | 97.3+/-6.4 | 98.9+/-3.8 | 99.5+/-2.8 |
|  | 3 h | 97.2+/-1.9 | 93.4+/-2.4 | 85.1+/-2.2 | 105.8+/-9.7 | 93.8+/-2.7 | 93.7+/-2.7 | 94.8+/-1.6 | 91.6+/-2.4 |
|  | 1 day | 113.1+/-15.9 | 85.2+/-14.8 | 80.6+/-5.8 | 93.9+/-5.0 | 96.4+/-3.6 | 94.6+/-6.0 | 94.6+/-0.8 | 90.7+/-2.9 |
|  | 5 days | 105.6+/-6.4 | 67.6+/-5.8 | 79.9+/-2.6 | 101.1+/-8.1 | 98.2+/-1.8 | 91.8+/-2.9 | 95.4+/-1.9 | 104+/-5.8 |
|  | 6 days | 100+/-0.9 | 42.3+/-1.6 | 82.4+/-7.6 | 102.0+/-7.0 | 97.3+/-0 | 92+/-3.7 | 94.8+/-0.5 | 93.8+/-3.1 |
| Freeze non-reconstituted samples  (-20 ^o^C) | 2 weeks | 111.2+/-1.9 | 75.6+/-1.8 | 86.1+/-3.7 | 77.1+/-0.3 | 94.6+/-0.9 | 94+/-2 | 92.4+/-1.9 | 86.7+/-0.5 |
|  | 4 weeks | 74.8+/-0.7 | 79.8+/-2.8 | 93.6+/-3.6 | 78.6+/-6.4 | 74.1+/-1.8 | 94.5+/-2.1 | 84.5+/-4.3 | 96+/-5.2 |
|  | 6 weeks | 86.9+/-0.9 | 94+/-16.7 | 94.5+/-0.8 | 81.9+/-9.5 | 70.5+/-3.6 | 95.4+/-4 | 87.5+/-3.3 | 92.2+/-2.5 |
|  | 2 months | 77.6+/-0.2 | 117.4+/-8.5 | 89.4+/-2.4 | 78.8+/-2.8 | 83+/-3.6 | 94+/-5.6 | 98.4+/-3.5 | 88+/-3.1 |
| Freezer (-20 ^o^C) | 4 weeks | 80.1+/-0.9 | 84.9+/-3.2 | 90.8+/-3.7 | 86.3+/-3.9 | 74.1+/-1.8 | 93.3+/-3.4 | 110.6+/-4.6 | 103.8+/-4 |
|  | 6 weeks | 83.2+/-1.9 | 107.7+/-5.1 | 92.8+/-4.9 | 85.8+/-2.8 | 70.5+/-1.8 | 91.4+/-4.8 | 114.9+/-3.3 | 92.8+/-3.7 |
|  | 2 months | 80.4+/-0.9 | 106.3+/-4.6 | 91+/-5.1 | 85.3+/-0.9 | 92.9+/-0.9 | 105+/-1.7 | 111.1+/-7.3 | 87.2+/-3.8 |
|  | 3 months | 96.2+/-7.9 | 113.3+/-4.4 | 87.7+/-9.3 | 93.9+/-2.8 | 87.7+/-14.3 | 109.6+/-4.7 | 93+/-1.9 | 88.8+/-3.3 |

The stability of the eight compounds with an associated deuterated standard was examined. For samples stored in the freezer for up to three months, the stability varied from around 1% to 30% in relation to the zero-time sample. Similarly, for samples prepared on the same day but reconstituted at different time intervals within the zero point to two months, the stability varied between 2% and 30%. In the case of samples stored at room temperature for a period of one hour to six days, the stability ranged from 1% to 20%, except for cocaine. Additionally, all other compounds that did not have an associated internal standard remained detectable when stored at room temperature for up to five days, as well as for samples stored before and after sample preparation in the freezer at -20 ^o^C.

The testosterone to epitestosterone (T/E) ratio was determined in triplicates of a pooled urine sample made from both female and male urine obtained at different times during the day and was spiked in four levels (1:1, 4:1, 6:1, and 8:1) of testosterone and epitestosterone.

The testosterone-to-epitestosterone ratio spiked in the urine samples and reflected the theoretical values. Moreover, the 1:1 spike was found to be 1.7 and the unspiked sample was 0.85. For data, please see Supplementary document Table 4. The fact that the unspiked sample did not precisely measure 1 was expected, considering that the urine used is a mixture of both female and male urine. Additionally, the T/E ratio in women can vary depending on the phase of their menstrual cycle [2].

**Supplementary document Table 4**: Results from the testing of the testosterone to epitestosterone ratio

| **Sample, T/E ratio** | **T/E ratio (mean ± SD)** |
| --- | --- |
| Unspiked urine | 0.85 ± 0.06 |
| Spiked samples, 1:1 | 1.73 ± 0.11 |
| Spiked samples, 4:1 | 3.99 ± 0.07 |
| Spiked samples, 6:1 | 6.08 ± 0.24 |
| Spiked samples, 8:1 | 7.30 ± 0.30 |

**References**

1. Harris, D.C., *Quantitative Chemical Analysis*. 2010: W. H. Freeman.

2. Schulze, J., et al., *Urinary steroid profile in relation to the menstrual cycle.* Drug Test Anal, 2021. **13**(3): p. 550-557.
